# The role of CXCL2-mediated crosstalk between tumor cells and macrophages in *Fusobacterium nucleatum*-promoted oral squamous cell carcinoma progression

**DOI:** 10.1038/s41419-024-06640-7

**Published:** 2024-04-18

**Authors:** Fujiao Nie, Jie Zhang, Haoyang Tian, Jingjing Zhao, Pizhang Gong, Huiru Wang, Suli Wang, Pishan Yang, Chengzhe Yang

**Affiliations:** 1 Department of Periodontology, School and Hospital of Stomatology, Cheeloo College of Medicine, Shandong University & Shandong Key Laboratory of Oral Tissue Regeneration & Shandong Engineering Research Center of Dental Materials and Oral Tissue Regeneration & Shandong Provincial Clinical Research Center for Oral Diseases, Jinan, Shandong China; 2 Advanced Medical Research Institute, Shandong University, Jinan, Shandong China; 3 Department of Oral and Maxillofacial Surgery, Qilu Hospital of Shandong University, Jinan, Shandong China

**Keywords:** Cancer microenvironment, Chemokines, Oral cancer

## Abstract

Dysbiosis of the oral microbiota is related to chronic inflammation and carcinogenesis. *Fusobacterium nucleatum* (*Fn*), a significant component of the oral microbiota, can perturb the immune system and form an inflammatory microenvironment for promoting the occurrence and progression of oral squamous cell carcinoma (OSCC). However, the underlying mechanisms remain elusive. Here, we investigated the impacts of *Fn* on OSCC cells and the crosstalk between OSCC cells and macrophages. 16 s rDNA sequencing and fluorescence in situ hybridization verified that *Fn* was notably enriched in clinical OSCC tissues compared to paracancerous tissues. The conditioned medium co-culture model validated that *Fn* and macrophages exhibited tumor-promoting properties by facilitating OSCC cell proliferation, migration, and invasion. Besides, *Fn* and OSCC cells can recruit macrophages and facilitate their M2 polarization. This crosstalk between OSCC cells and macrophages was further enhanced by *Fn*, thereby amplifying this positive feedback loop between them. The production of CXCL2 in response to *Fn* stimulation was a significant mediator. Suppression of CXCL2 in OSCC cells weakened *Fn*’s promoting effects on OSCC cell proliferation, migration, macrophage recruitment, and M2 polarization. Conversely, knocking down CXCL2 in macrophages reversed the *Fn*-induced feedback effect of macrophages on the highly invasive phenotype of OSCC cells. Mechanistically, *Fn* activated the NF-κB pathway in both OSCC cells and macrophages, leading to the upregulation of CXCL2 expression. In addition, the SCC7 subcutaneous tumor-bearing model in C3H mice also substantiated *Fn*’s ability to enhance tumor progression by facilitating cell proliferation, activating NF-κB signaling, up-regulating CXCL2 expression, and inducing M2 macrophage infiltration. However, these effects were reversed by the CXCL2-CXCR2 inhibitor SB225002. In summary, this study suggests that *Fn* contributes to OSCC progression by promoting tumor cell proliferation, macrophage recruitment, and M2 polarization. Simultaneously, the enhanced CXCL2-mediated crosstalk between OSCC cells and macrophages plays a vital role in the pro-cancer effect of *Fn*.

## Introduction

Oral squamous cell carcinoma (OSCC) is a prevalent malignant tumor in the head and neck region, exhibiting a high incidence rate with over 300,000 new cases reported annually worldwide [1, 2]. The five-year survival rate of OSCC patients is only 50%-60% due to its high invasion and metastasis rate [3–5]. Multiple risk factors have been identified, including betel nut chewing, excessive alcohol consumption, smoking, and human papillomavirus (HPV) infection [2]. Periodontal pathogens and other oral microbiomes have recently been recognized as independent risk factors [6]. Some evidence indicates that the imbalance of oral microbiota plays a pivotal role in the onset, progression, and prognosis of OSCC, thereby offering potential biomarkers for OSCC [7–9]. The oral microbiota can elicit chronic inflammation, generate inflammatory mediators and carcinogenic agents, and influence cell proliferation and apoptosis. In addition, inflammatory reactions can attract immune cells to infiltrate the oral mucosa and modulate the immune microenvironment.

*Fusobacterium nucleatum* (*Fn*), a prevalent commensal microbe in the oral cavity, is recognized as a core species of plaque biofilms and a critical pathogenic bacterium in chronic periodontitis [10]. This anaerobic microbe exhibits invasiveness, adhesion, and pro-inflammatory properties, and can interact with *Porphyromonas gingivalis* to promote the progression of inflammation in periodontitis [11, 12]. In addition, *Fn* exhibits a close correlation with OSCC, with notably elevated abundance observed in OSCC tissues in contrast to non-cancerous tissues [13–15]. However, the etiological association of *Fn* in OSCC requires further validation through extensive studies.

Tumor-associated macrophages (TAMs) constitute the predominant immune cell population in the tumor microenvironment (TME) and have a pivotal influence on tumor progression [16]. It has been found that macrophages and tumor cells exhibit close spatial connections and engage in multi-directional intercellular communication [17]. Recruitment and polarization of TAMs are orchestrated by cytokines and chemokines derived from both tumor and host [18]. In parallel, TAMs can produce a range of cytokines and chemokines that promote tumor advancement and invasion while eliciting immunosuppressive responses [19–23]. Chemokines exert direct regulatory effects on various tumor cell processes, including proliferation, angiogenesis, migration, invasion, and chemotherapy resistance [24]. They also play a significant role in the recruitment and activation of immune cells in the TME during tumor progression [25–29]. This suggests the existence of a reciprocal crosstalk between the macrophages and tumor cells, potentially mediated by chemokines, leading to the establishment of a pro-cancer feedback loop [22, 30]. Furthermore, research indicated that *Fn* can enhance macrophage M2 polarization and augment the malignancy of tumor cells [28, 31–34]. However, whether *Fn* can amplify the bidirectional crosstalk between tumor cells and macrophages in OSCC remains poorly defined, and the critical mediating factors require further elucidation.

Here, we investigated *Fn*’s effects on bidirectional crosstalk between OSCC cells and macrophages and its underlying molecular mechanism. Interestingly, our findings revealed that *Fn* facilitates the secretion of chemokine CXCL2 by OSCC cells and macrophages by activating the NF-κB pathway. This intricate crosstalk between OSCC cells and macrophages, facilitated by CXCL2, leads to OSCC cell migration, macrophage recruitment, and M2 polarization. Consequently, the macrophage-OSCC cell interactions promoted by *Fn* established a positive feedback loop, ultimately reinforcing tumor progression. These findings enhance our understanding of molecular mechanisms underlying *Fn*-mediated tumor progression and immunosuppression, and provide a novel predictive biomarker and feasible therapeutic target for OSCC.

## Results

### Enrichment of *Fn* and macrophages in OSCC

**Fig. 1:**
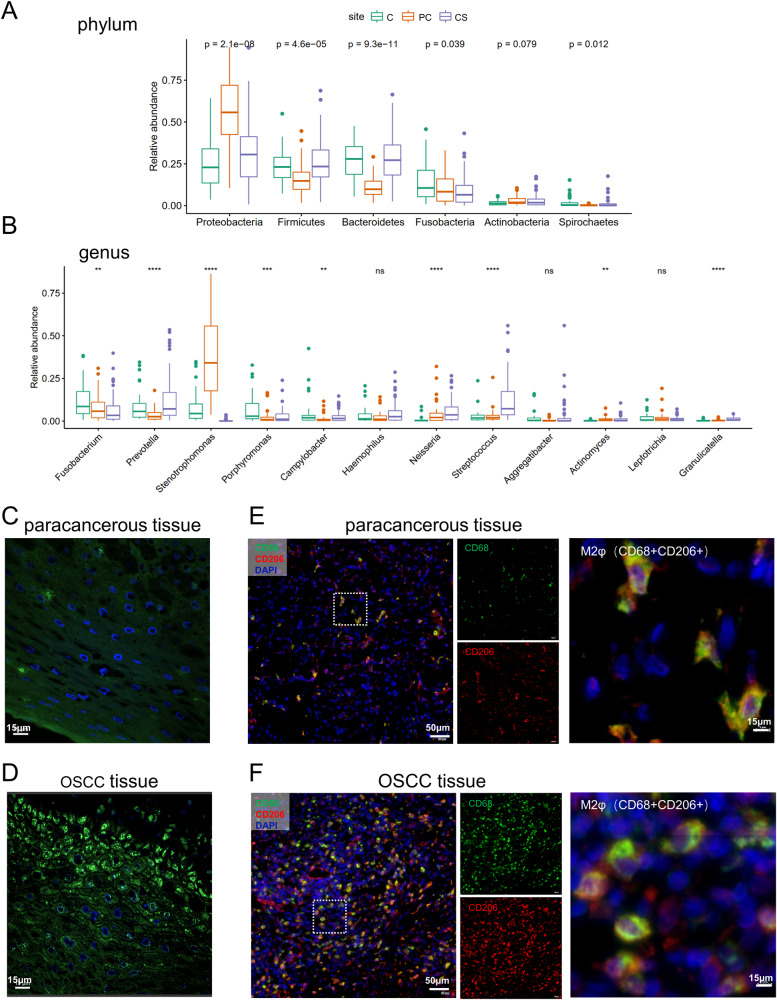
The *Fn* and M2 macrophages were enriched in OSCC tissues compared with paracancerous tissues. **A**, **B** Bacterial community composition at the phylum (**A**) and genus (**B**) level. **C**, **D** Detection of *Fn* by FISH in paracancerous tissues (**C**) and OSCC tissues (**D**). **E**, **F** IF analysis of the expression of CD68 and CD206 in paracancerous tissues (**E**) and OSCC tissues (**F**). CD68+ was used to label human macrophages, and CD68 + CD206+ to label human M2 macrophages.

We first demonstrated the existence of *Fn* and the phenotype of macrophages in clinical OSCC samples. 16s rDNA sequencing showed that *Fusobacteria* and *Fusobacterium* exhibit a notable enrichment in tumor tissues (C) compared to both the tumor surface (CS) and paracancerous tissues (PC) at the phylum (Fig. 1A) and genus (Fig. 1B) level. FISH analysis revealed a greater abundance of *Fn* in tumor tissues (Fig. 1D) compared to adjacent non-tumor tissues (Fig. 1C). In addition, the immunofluorescence (IF) staining demonstrated an increased presence of macrophages, particularly M2 macrophages, within the tumor tissues (Fig. 1E, F).

### Both *Fn* and macrophages contribute to the proliferation and migration of OSCC cells, and *Fn*-challenged macrophages amplify this pro-tumor effect

Before establishing the co-culture systems, the optimal multiplicity of infection (MOI) of *Fn* was determined through CCK8 (Figs. S1A, S3A, and S3C) and living death staining (Fig. S1B). According to the experimental results, the MOI 100 was selected for the subsequent experiments in human tongue squamous cell carcinoma cell line cal27 and murine head and neck squamous cell carcinoma (HNSCC) cell line SCC7. MOI 20 was used in human monocyte line THP-1 cells (hMφ), and MOI 10 was used in murine macrophage cell line RAW264.7 (mMφ). The THP-1 cells were pretreated with PMA to induce differentiation into macrophages in all experiments. Subsequently, two co-culture models were established: cal27 and THP-1 cells (model 1), or SCC7 and RAW264.7 cells (model 2), with or without *Fn*, based on the selected optimal MOI to investigate the impact of *Fn*, macrophages, and *Fn*-challenged macrophages on proliferation, migration, and invasion of OSCC cells. Moreover, according to Fig. S2, Fig. S3B, and S3D, the SCC7 and RAW264.7 cells were infected with *E. coli* with MOI 10 as a control in model 2.

**Fig. 2:**
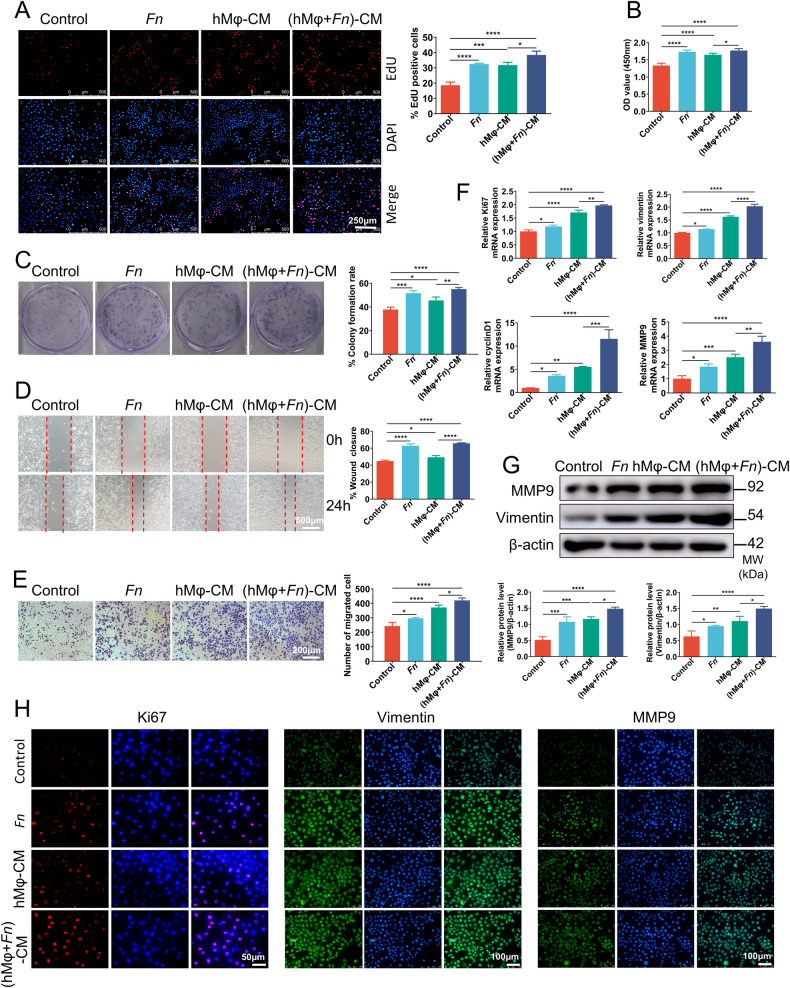
Both *Fn* and macrophages contribute to the proliferation and migration of cal27 cells, and *Fn*-challenged macrophages amplify this pro-tumor effect. **A**–**C** The proliferation ability of cal27 cells treated with *Fn*, hMφ-CM, and (hMφ + *Fn*)-CM was assessed using Edu assays (**A**), CCK8 assays (**B**), and colony formation assays (**C**). **D**, **E** The mobility and migration capacity of cal27 cells was detected by wound healing assay (**D**) and transwell assay (**E**). **F** qRT-PCR was performed to detect Ki67, vimentin, cyclinD1, and MMP9 mRNA expression in cal27 cells in response to different stimulation. **G** Western blot analysis of vimentin and MMP9 in cal27 cells. **H** IF assay of ki67, vimentin, and MMP9 in cal27 cells. *P < 0.05, **P < 0.01, ***P < 0.001, and ****P < 0.0001, ns, no significant.

The results of Edu (Fig. 2A) and CCK8 (Fig. 2B) assays demonstrated that both *Fn* and THP-1-conditioned medium (hMφ-CM and hMφ + *Fn*-CM) could enhance the proliferation of the cal27 cells compared to the control group. Furthermore, the pro-proliferation effect of *Fn*-stimulated hMφ-CM (hMφ + *Fn*-CM) was more significant than hMφ-CM. These results were further supported by the colony formation assay, which revealed that the cal27 cells in the *Fn* and conditioned medium groups exhibited a greater number and larger size of cell clones in comparison to the control group, with the (hMφ + *Fn*)-CM demonstrating a more pronounced effect compared to hMφ-CM (Fig. 2C). *Fn*, RAW264.7-conditioned medium (mMφ-CM) and *Fn*-stimulated mMφ-CM (mMφ+*Fn*-CM) also exhibited significant pro-proliferation effect on SCC7 cells relative to the blank control and *E.coli* control (Figs. S4A, S4B and S4C). This suggests that both *Fn* and macrophages contribute to the proliferation of OSCC cells, and *Fn* stimulation amplifies the promoting proliferation effect of macrophages on OSCC cells.

Transwell and scratch tests were employed to assess the cal27 cell migration abilities following co-incubation with *Fn*, hMφ-CM, and (hMφ + *Fn*)-CM. As shown in Fig. 2D, E, *Fn* infection and conditioned medium stimulation enhanced the migration abilities of cal27 cells. Notably, the effect observed in the (hMφ + *Fn*)-CM group was more pronounced compared to the hMφ-CM group. Furthermore, the findings from PCR, western blot, and IF experiments consistently supported the above results. The *Fn* and conditioned medium groups demonstrated upregulation of the mRNA expression of ki67, vimentin, cyclinD1, and MMP9 (Fig. 2F), along with an increase in the protein levels of ki67, vimentin, and MMP9 (Fig. 2G, H). Similar results were also demonstrated in another co-culture system (SCC7 and RAW264.7 cells). *Fn*, mMφ-CM, and (mMφ+*Fn*)-CM presented significant pro-invasion and migration potentials for SCC7 cells relative to the blank control and *E.coli* control (Fig. S4D and S4E). These findings indicate that *Fn* and macrophages have the potential to promote the proliferation and migration of OSCC cells directly, and *Fn* augments the promoting effect of macrophages on the aggressive phenotype of OSCC cells.

### Both *Fn* and OSCC cells recruit macrophages and promote M2 macrophage polarization, and *Fn*-challenged OSCC cells augment the effects

**Fig. 3:**
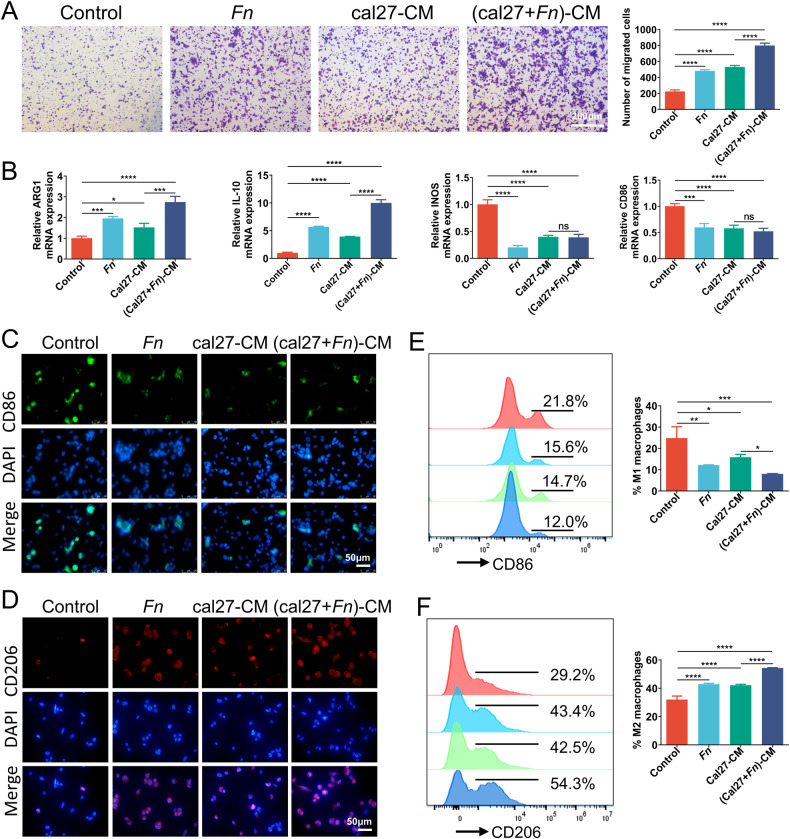
Both *Fn* and OSCC cells recruit macrophages and promote M2 macrophage polarization, and *Fn*-challenged tumor cells augment the effects. **A** Transwell assay assessed the capacity of *Fn* and cal27-conditioned medium to recruit THP-1 cells. **B** The effects of *Fn* and cal27-conditioned medium on M2 polarization of THP-1 cells were evaluated by qRT-PCR. **C**, **D** IF assays of THP-1 cells. **E**, **F** Flow cytometry assays of THP-1 cells. *P < 0.05, **P < 0.01, ***P < 0.001, and ****P < 0.0001, ns, no significant.

To investigate the effect of *Fn* and OSCC cells on macrophage recruitment and polarization, co-culture models incorporating macrophages and OSCC cells with *Fn* as stimulators were employed. Results showed that the presence of *Fn* and OSCC-conditioned medium elicited a recruitment effect on THP-1 (Fig. 3A) and RAW264.7 cells (Fig. S5A), with the recruitment effect of OSCC-conditioned medium being more pronounced when stimulated by *Fn*.

Then, to probe the role of *Fn* and OSCC cells in the polarization of macrophages, we utilized *Fn* and cal27-CM to stimulate THP-1 cells (model 1) and used *Fn* and SCC7-CM to incubate RAW264.7 cells (model 2). In model 1, the results of qRT-PCR assay indicated that all the experimental groups exhibited a decreased mRNA expression level of M1 marker (iNOS, CD86) and an increased mRNA expression level of M2 marker (IL-10, ARG1) compared to the control group, among which the impact of (cal27+*Fn*)-CM on M2 polarization was found to be more pronounced than cal27-CM (Fig. 3B). A similar trend was observed in IF investigations (Fig. 3C,D) and flow cytometry (Fig. 3E, F), wherein (cal27+*Fn*)-CM exhibited the most potent ability to enhance the expression of CD206, M2 marker for THP-1 cells, while concurrently inhibiting the expression of CD86, an M1 marker. The co-culture model of SCC7 and RAW264.7 cells showed similar results. IF (Fig. S5B and S5C) and flow cytometry (Fig. S5D and S5E) revealed that *Fn* and SCC7 cells suppressed M1 polarization while enhancing M2 polarization. Moreover, *Fn* further potentiated the capacity of SCC7 cells to promote M2 macrophage polarization. These findings prove that both *Fn* and OSCC cells can induce M2 macrophage polarization, with *Fn* additionally strengthening this capability of OSCC cells.

### Chemokine CXCL2 mediates *Fn*-intensified crosstalk between OSCC cells and macrophages

**Fig. 4:**
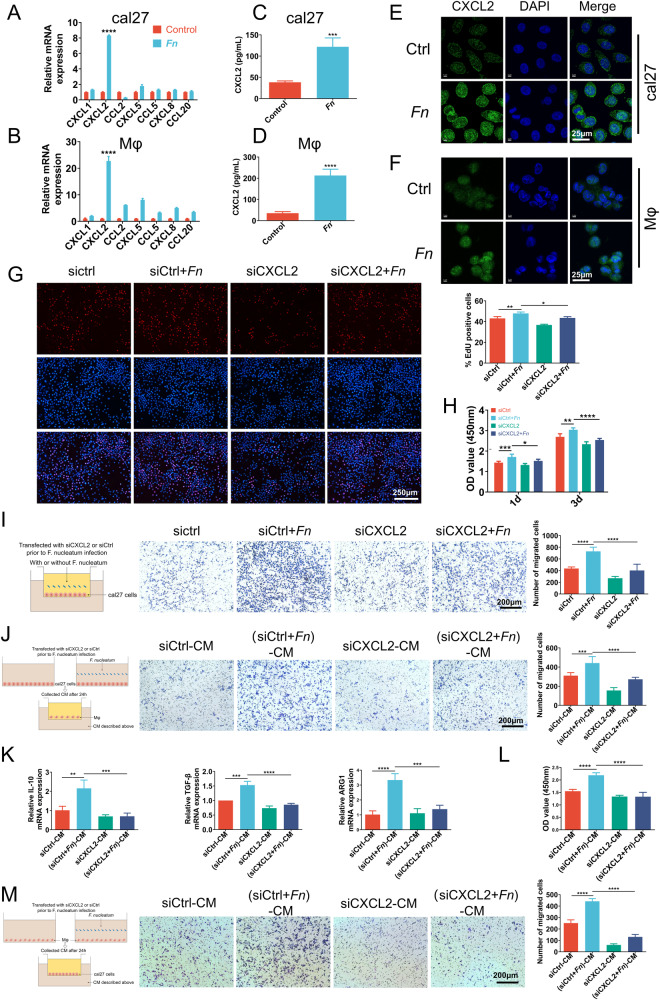
Chemokine CXCL2 mediates *Fn*-intensified crosstalk between OSCC cells and macrophages. **A**, **B** qRT-PCR analysis shows the expression of chemokine in cal27 (**A**) and THP-1 cells (**B**), with stimulation by *Fn* or without stimulation. **C**, **D** ELISA assay for CXCL2 in conditioned medium of cal27 (**C**) and THP-1 cells (**D**). **E**, **F** IF staining for CXCL2 protein expression in cal27 (**E**) and THP-1 cells (**F**). **G**–**I** Pretreated with CXCL2 siRNA or control siRNA, cal27 cells were incubated with or without *Fn* for 24 h. The proliferation ability was detected by Edu assays (**G**) and CCK8 assays (**H**), and the migratory ability of cal27 cells was detected by transwell assays (**I**). **J**, **K** Cal27 cells were pretreated with CXCL2 or control siRNA, followed by incubation with or without *Fn* for 24 h. The conditioned medium was collected to determine its impact on the macrophage recruitment (**J**) and the polarization towards the M2 phenotype (**K**). **L** CCK8 assays were performed to evaluate the impact of CXCL2 down-regulation in the macrophage-conditioned medium on the proliferation of cal27 cells. **M** Transwell assays were performed to evaluate the impact of CXCL2 down-regulation in the macrophage-conditioned medium on the migration of cal27 cells. *P < 0.05, **P < 0.01, ***P < 0.001, and ****P < 0.0001, ns, no significant.

Chemokines directly affect non-immune cells within the TME, such as tumor cells and vascular endothelial cells, thereby influencing tumor cell proliferation, tumor stem cell characteristics, and the capacity for metastasis and invasion. Besides, chemokines also facilitate the recruitment of distinct immune cell subsets and modulate the cellular immune response against tumors. Reports indicate that microorganisms, such as *Fn* and *Porphyromonas gingivalis*, can stimulate tumor cells or immune cells to secrete chemokines, thereby facilitating the malignant advancement of tumors [22, 28, 35–37]. Thus, chemokines are supposed to mediate *Fn*-intensified crosstalk between OSCC cells and macrophages. To testify to this hypothesis and ascertain the critical chemokine in this process, we first examined the expression levels of several chemokines (CXCL1, CXCL2, CCL2, CXCL5, CCL5, CXCL8, and CCL20), which have been extensively investigated in prior scholarly works [22, 23, 28, 37–39], through qRT-PCR analysis. We found that CXCL2 exhibited the highest expression levels in cal27 cells (Fig. 4A) and THP-1 cells (Fig. 4B) following stimulation with *Fn*. This outcome was further corroborated by the enzyme-linked immunosorbent assay (ELISA) (Fig. 4C, D) and IF staining (Fig. 4E, F), suggesting that *Fn* improves CXCL2 expression and secretion in cal27 cells and THP-1 cells. Similar results were obtained in the co-culture system of SCC7 and RAW264.7 cells (Fig. S6).

Then, gene silencing in cal27 cells was used to evaluate the role of CXCL2 in the *Fn*-pro-cancer effect and *Fn*-amplifying macrophage recruitment and M2 polarization effects. As demonstrated in Fig. S8A and S8B, the silencing efficacy was confirmed, and siRNA-3 was selected for subsequent experiments. As indicated by Edu (Fig. 4G) and CCK8 (Fig. 4H) results, the inhibition of CXCL2 in cal27 cells resulted in a reversal of the promoting-proliferation effect of *Fn*. In addition, transwell and scratch assays showed that the promoting-migration effect of *Fn* was attenuated upon silencing CXCL2 (Fig. 4I and Fig. S9A). In addition, following CXCL2 knockdown in cal27 cells, the promotion effects of (cal27+*Fn*)-CM on recruitment (Fig. 4J) and M2 polarization (Fig. 4K) of THP-1 cells were also attenuated. These results suggest that CXCL2 in cal27 cells mediates the pro-cancer, enhanced macrophage recruitment, and M2 polarization effects of *Fn*.

Finally, to evaluate the role of CXCL2 in the pro-cancer activity of macrophages, the CXCL2 gene in THP-1 cells was silenced. Silencing efficiency was verified, as shown in Figs. S8A and S8C, and the subsequent experiments were performed using siRNA-3. Subsequently, the macrophage-conditioned medium was obtained with or without *Fn* stimulation. As shown in Fig. 4L, M, and S9B, the potency of (hMφ + *Fn*)-CM in enhancing the proliferation (Fig. 4L) and migration (Fig. 4M and Fig. S9B) of cal27 cells was diminished upon silencing macrophages’ CXCL2. These results imply that CXCL2 mediates the *Fn*-reinforced pro-cancer effect of macrophages.

### *Fn* regulates CXCL2 through the NF-κB signaling pathway

**Fig. 5:**
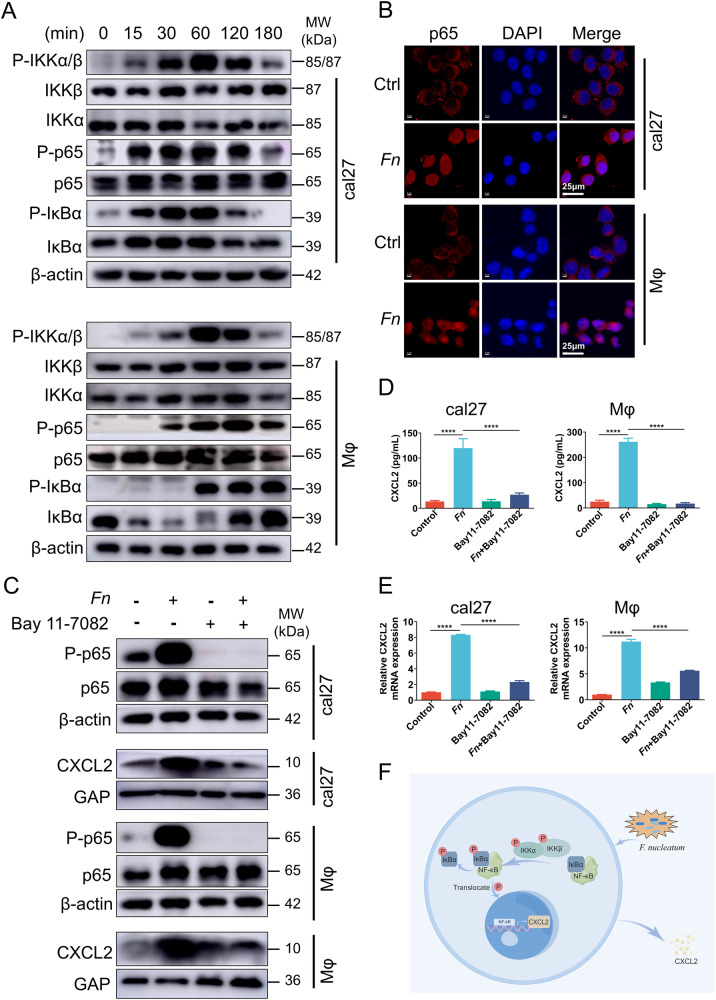
*Fn* regulates CXCL2 through the NF-κB signaling pathway. **A** Western blot analysis of NF-κB pathway members expression in cal27 and THP-1 cells cocultured with *Fn* for 0, 15, 30, 60, 120, and 180 mins. **B** IF assay was used to visualize the nucleus translocation of p65 with the treatment of *Fn* in cal27 and THP-1 cells. **C** Western blot analysis of CXCL2 level in cal27 cells and THP-1 cells pretreated with or without inhibitors Bay 11-7082 for 60 min and then cocultured with or without *Fn* for 48 h. **D** ELISA analysis of CXCL2 levels in the conditioned medium of cal27 (left) and THP-1 cells (right) pretreated with or without inhibitors Bay 11-7082 for 60 min and then cocultured with or without *Fn*. **E** qRT-PCR analysis for CXCL2 levels in cal27 (left) and THP-1 cells (right). **F** Schematic diagram of the NF-κB pathway. *P < 0.05, **P < 0.01, ***P < 0.001, and ****P < 0.0001, ns, no significant.

To further study the upstream regulators that might transmit signals to the CXCL2, we explored possible pathways involved in *Fn*-mediated CXCL2 secretion by macrophages and OSCC cells. NF-κB pathway and its upstream regulators IKK and IκBα, which have been demonstrated to be activated by *Fn* in the progression of colorectal cancer and esophageal squamous cell carcinoma [31, 40–43], were analyzed in the present study. Western blot analysis revealed that in cal27 and THP-1 cells, *Fn* treatment led to a significant upregulation of p-IKKα/β and p-p65 while simultaneously causing a reduction of total IκBα in a time-dependent manner (Fig. 5A). These findings suggest that *Fn* induces the activation of IKKα/β and the degradation of NF-κB-bound IκBα, ultimately resulting in the activation of NF-κB (Fig. 5F). The result of IF also showed that the nuclear translocation of p65 after the stimulation with *Fn* (Fig. 5B). These findings suggest that *Fn* triggers the activation of NF-κB signaling.

To further confirm the correlation between the activation of the NF-κB signaling and the secretion of CXCL2, cal27, and THP-1 cells were treated with the NF-κB pathway inhibitor Bay11-7082 alone or together with *Fn*. Upon the administration of the Bay11-7082, the *Fn*-induced upregulation of p-p65 was effectively inhibited (Fig. 5C). Furthermore, CXCL2 protein expression (Fig. 5C) and secretion (Fig. 5D) and its gene expression (Fig. 5E) results suggested the level of CXCL2 up-regulated by *Fn* was suppressed by NF-κB pathway inhibitor. These findings suggest the involvement of the NF-κB pathway in *Fn*-induced upregulation of CXCL2 in oral squamous cell carcinoma (Fig. 5F).

### *Fn* facilitates the progression of OSCC and promotes the infiltration of M2 macrophages through the CXCL2 axis in mice

**Fig. 6:**
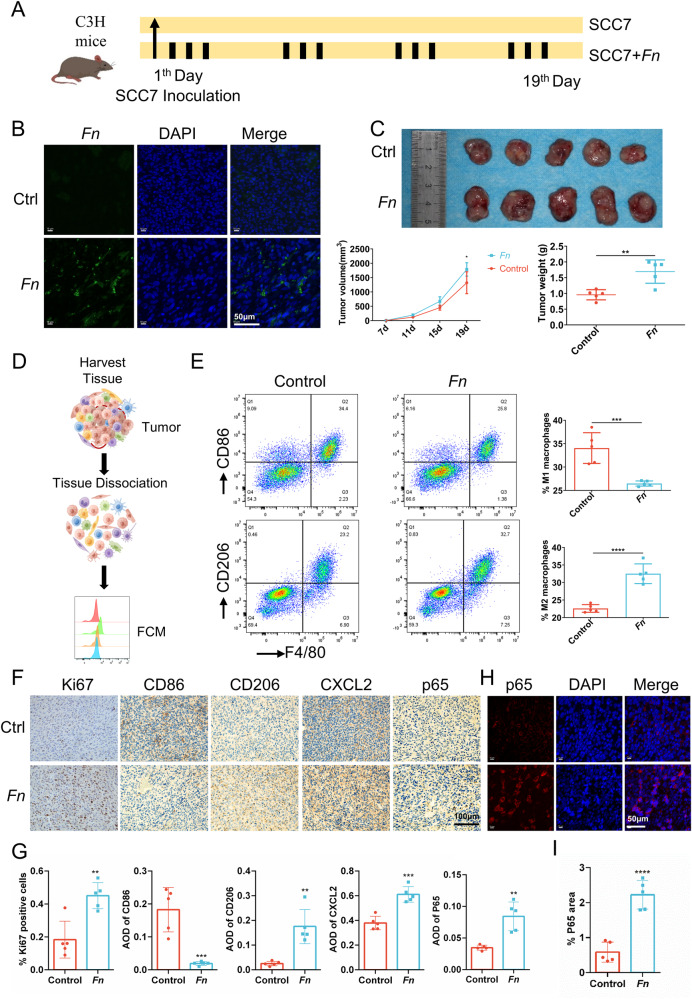
*Fn* facilitates the progression of OSCC and promotes the infiltration of M2 macrophages in mice. **A** Schematic diagram of mice modeling. **B** Detection of *Fn* by FISH in SCC7 tumors. **C** Photographs of tumors and tumor growth curves and weights. **D** Schematic for tumor dissociation. **E** Representative flow cytometry plots of tumor-infiltrating M1 macrophages (F4/80 + CD86+) and M2 macrophages (F4/80 + CD206+). **F** Representative IHC staining of Ki67, CD86, CD206, CXCL2 and p65. **G** Quantification of IHC staining for Ki67, CD86, CD206, CXCL2 and p65. **H** IF staining of p65 in SCC7 tumors. **I** Quantitative analysis of IF staining. *P < 0.05, **P < 0.01, ***P < 0.001, and ****P < 0.0001, ns, no significant.

To further investigate the potential tumor-promoting effect of *Fn* in OSCC, we established an in vivo subcutaneously transplanted tumor model using the SCC7 cells in C3H mice and intervened by administering *Fn* through multi-point injection (Fig. 6A). The existence of *Fn* in *Fn* group was verified by FISH detection (Fig. 6B). The *Fn* group exhibited increased tumor weight and volume compared to the control group (Fig. 6C). Flow cytometry analysis of tumor tissues revealed a decrease in M1 macrophages, accompanied by an increase in M2 macrophages in the *Fn* group relative to the control group (Fig. 6D, E). The immunohistochemical (IHC) findings demonstrated more prominent expression of Ki67, CD206, CXCL2, and p65 while less expression of CD86 in the *Fn* group compared to the control group (Fig. 6F, G). IF staining of CD86 and CD206 indicated a significant increase in the CD206/CD86 ratio in the *Fn* group (Fig. S10). Translocation of p65 to the nucleus following *Fn* stimulation was demonstrated by IF staining (Fig. 6H, I). In addition, the tumor tissues were lysed, and the p-p65, p65, and CXCL2 levels were detected by western blot. It indicated that *Fn* activated NF-κB p65 signaling and promoted an upregulation of CXCL2 expression in tumor tissues (Fig. S11). These findings further imply that *Fn* facilitates tumor cell proliferation, macrophage recruitment, and M2 polarization.

To further elucidate the involvement of pivotal regulators CXCL2 in *Fn*-enhanced tumor growth and macrophage M2 polarization, we used a selective inhibitor SB225002 for CXCR2 (the receptor for chemokines CXCL2). As shown in Fig. S12A, SB225002 reversed the tumor‐promoting effect of *Fn*. In addition, it counteracted the promotion of macrophage polarization toward the M2 phenotype induced by *Fn* (Fig. S12B).

## Discussion

**Fig. 7:**
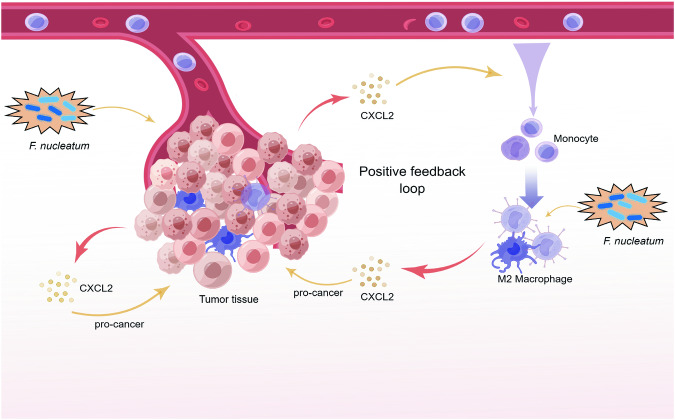
Schematic diagram of the *Fn* enhancing the CXCL2-mediated crosstalk between OSCC cells and macrophages. *Fn* was found to activate the self-reinforcing CXCL2 signaling loop, which governs the intricate interactions between OSCC cells and macrophages. This activation is deemed crucial in driving the progression of OSCC.

Tumor progression is a complicated process wherein tumor cells interact with TME for reciprocal promotion. This study discovered a novel pro-tumor mechanism of *Fn* in OSCC involving the enhanced crosstalk between tumor cells and macrophages. Specifically, we found that *Fn* triggers the activation of the NF-κB signaling pathway in OSCC cells and macrophages, leading to the secretion of CXCL2. This increase in CXCL2 within the TME facilitates the proliferation and migration of OSCC cells, as well as the recruitment and M2 polarization of macrophages. Overall, this CXCL2-mediated bidirectional crosstalk between OSCC and macrophages forms a positive feedback loop facilitated by *Fn* stimulation (Fig. 7).

In recent years, growing attention has been focused on the microorganisms’ involvement in the advancement of tumors. The microbiota affects the host’s immune system, and simultaneously, microbiota and the immune system modulate TME and influence tumor development [44]. *Fn*, a bridging microorganism involved in the formation and structural organization of oral multi-species biofilms, is mainly enriched in periodontitis microenvironment [11]. *Fn* has been reported to be enriched in colorectal cancer [45, 46], breast cancer [47], pancreatic cancer [48], esophageal cancer [49], and OSCC [13]. Its presence is associated with poor prognosis and treatment failure in some tumors [49–54]. The animal model also reveals that *Fn* accelerates tumor growth and metastatic progression [47, 55–57] while also facilitating the establishment of a tumor-permissive immune microenvironment [56]. These findings suggest an essential role of *Fn* in tumor progression, especially in colorectal cancer (CRC). However, *Fn*’s potential association with OSCC has received comparatively limited investigation [58, 59]. Considering the fact that CRC-associated *Fn* mainly originates from the oral microbiota [60], while *Fn* in oral microbiota has a stronger association with OSCC relative to CRC, it is urgent to further elucidate the specific role and underlying mechanism of *Fn* in OSCC progression.

As an essential component of the tumor immune microenvironment, macrophages make up 30 to 50% of solid tumor-infiltrating immune cells [61]. Macrophage-featured inflammation is recognized as the seventh hallmark of cancer [62]. Macrophages possess notable functional heterogeneity and adaptability, wherein their phenotype and function can be modulated in response to microenvironmental signals, forming a classically (M1) or alternatively (M2) activated state, with anti-tumor or pro-cancer properties, respectively [63–65]. Tumor cells play a crucial role in macrophage recruitment and M2 polarization [66], with the M2 subtype predominantly dominating the TAMs. Meanwhile, TAMs can promote tumor advancement and invasion, and induce immunosuppressive effects [19–23]. Thus, a reciprocal crosstalk occurs between the TAMs and tumor cells, facilitating tumor malignant behaviors and immune evasion [67]. To identify *Fn*’s potential and underlying mechanism in this bidirectional crosstalk of OSCC, we developed an in vitro co-culture model involving *Fn*, OSCC cells, and macrophages. Our findings indicate that both *Fn* and macrophages facilitate the proliferation and migration of OSCC cells. In addition, we observed that *Fn* and OSCC cells contribute to macrophage recruitment and M2 polarization. Notably, macrophages stimulated by *Fn* exhibit a more pronounced promoting effect on OSCC cells, whereas OSCC cells stimulated by *Fn* have a more evident impact on macrophage recruitment and M2 polarization compared to those not stimulated by *Fn*. The in vivo mice SCC7 tumor model also demonstrated that *Fn* could accelerate tumor growth and macrophage M2 polarization. These findings demonstrate that *Fn* amplifies the crosstalk between OSCC cells and macrophages, significantly contributing to the advancement in OSCC.

Recently, immune molecules (growth factor, inflammatory cytokines, and chemokines) derived from TAMs and TME have gained significant recognition as mutual mediators of signal transduction [68]. Notably, chemokines act as crucial messengers that mediate the crosstalk between tumor cells and TAMs via autocrine or paracrine pathways [18]. Chemokines (such as CCL2, CCL3, CCL5, CCL7, CCL15, CXCL8, and CXCL12) secreted by tumors and hosts can stimulate the recruitment and differentiation of monocytes into macrophages and contribute to the infiltration and immunosuppressive capabilities of macrophages in tumors [35, 69]. Meanwhile, macrophages can promote tumor invasion and metastasis through secretion of various cytokines (e.g., TGF-β, IL-10, arginase 1) and chemokines (e.g., CCL5, CCL17, CCL18, CCL22) [18, 22, 27, 70–73]. However, whether chemokines mediate *Fn*-enhanced bidirectional crosstalk between OSCC cells and macrophages remains unclear. To this end, we examined the impact of *Fn* on the secretion of chemokines by cal27 and THP-1 cells and identified CXCL2 as the most significantly altered chemokine following *Fn* stimuli among CXCL1, CXCL2, CCL2, CXCL5, CCL5, CXCL8, and CCL20.

CXCL2 is primarily synthesized by tumor cells, neutrophils, and macrophages [74]. Prior research has demonstrated a mediating role of CXCL2 in bidirectional crosstalk between tumor cells and TAMs. Bao et al. [75] revealed that the secretion of CXCL2 by transformed mesenchymal cells in CRC promotes M2 macrophage infiltration and lung metastases of tumor cells. Cai et al. [23] confirmed that CXCL2 secreted by M2-TAM can enhance gastrointestinal stromal tumor cells’ invasion, migration, and mesenchymal transformation. The high expression of CXCL2 can facilitate the metastasis of diverse malignant tumor cells [76, 77]. Besides, the suppression of CXCL2 in tumor cells impedes the invasive properties of osteosarcoma and breast cancer [78, 79]. The present study found that CXCL2 gene knockdown in cal27 cells not only attenuated the ability of *Fn* to enhance cal27 cell proliferation and migration, but also reduced *Fn*-strengthened macrophage recruitment and M2 polarization effects of cal27-conditioned medium. CXCL2 gene knockdown in THP-1 cells reversed the *Fn*-intensified impact of THP-1-conditioned medium on cal27 cell proliferation and migration. In summary, this study indicates that CXCL2 may function as a mediator between OSCC progression and macrophage infiltration, consistent with the other studies [23, 75]. Moreover, *Fn* can reinforce bidirectional crosstalk between OSCC cells and macrophages by up-regulating CXCL2 expression in two kinds of cells. The in vivo experiments also verified that the *Fn*-induced pro-tumor effect was attenuated after blocking the CXCL2 receptor (CXCR2).

*Fn* can activate the NF-κB pathway, resulting in the upregulation of pro-inflammatory cytokines and chemokines, which leads to the formation of a pro-inflammatory TME and the selective recruitment of bone marrow-derived immune cells, including bone marrow-derived suppressor cells (MDSCs), tumor-associated neutrophils (TANs), TAMs, and dendritic cells (DCs) [10, 28]. To explore the upstream regulation mechanism of *Fn*-enhanced CXCL2 expression, our investigation centered on the potential involvement of NF-κB signaling. The in vitro and in vivo investigation showed that *Fn* infection could activate the NF-κB pathway. The activated NF-κB pathway actively contributed to the upregulation of CXCL2, thereby facilitating the bidirectional crosstalk between OSCC cells and macrophages.

In summary, we elucidated that the self-reinforcing CXCL2 signaling loop activated by *Fn* stimulation, governing the crosstalk between OSCC cells and macrophages, plays a crucial role in driving OSCC progression. Our findings offer a novel perspective on the potential therapeutic targeting of chemokines and immune dynamic balance for OSCC. However, the existing research on the intricate upstream and downstream signaling networks of CXCL2 needs to be improved, necessitating further investigation to augment our comprehension of tumor progression.

## Materials and methods

### 16S rDNA gene sequencing

Methods of sample collection, DNA extraction and amplification, and 16S ribosomal DNA sequencing have been previously described [80].

### Clinical samples

A total of ten tumor tissues and ten adjacent normal tissues (paracancerous tissues) were collected from patients with OSCC at the first cancer diagnosis, who did not receive preoperative anticancer treatment and did not use antibiotic treatment for at least three months. Written informed consent was obtained from all participants. The study was approved by the Ethics Committee of the Hospital of Stomatology, Shandong University (No.20230366). After collection, routine tissue dehydration, transparention, wax immersion, and embedding were performed.

### Fluorescence in situ hybridization (FISH)

*Fn* detection was conducted by FISH on 4 mm paraffin-embedded tissue sections. The FISH kit and *Fn*-targeted probe (5′-CGCAATACAGAGTTGAGCCCTGC-3ʹ) were purchased from Guangzhou EXON Biological Technology company (Guangzhou, China). The sections were deparaffinized, rehydrated, and hybridized with the probe following the manufacturer’s instructions. Confocal images were captured using a spinning disk confocal microscope (Andor Technology).

### Bacterial strain and cell culture

*Fn* strain (ATCC 25586) and *E. coli* strain were provided by the Shandong Key Laboratory of Oral Tissue Regeneration (Jinan, China). *Fn* was cultured in brain heart infusion (BHI) broth supplemented with hemin (5 mg/l) and Vitamin K1 (1 mg/l) under anaerobic conditions at 37 °C. After incubating overnight, bacterial colony-forming units (CFUs) were measured at OD600, and bacterial concentrations were adjusted subsequently. Then, bacteria were washed with PBS and used for co-culture experiments. The *E. coli* strains were cultivated in lysogeny broth (LB) at 37 °C.

Cal27, THP-1, SCC7, and RAW264.7 cells were purchased from the Cell Bank of the Chinese Academy of Sciences (Shanghai, China). The cal27, SCC7, and RAW264.7 cells were cultured in DMEM (Gibco, Grand Island, NY) containing 10% fetal bovine serum (FBS, Gibco) and 1% penicillin-streptomycin. THP-1 cells were cultured in RPMI 1640 medium (Gibco) containing 10% FBS and 0.05 mM β-mercaptoethanol (Sigma, St. Louis, MO). All the cells were cultured at 37 °C in a humidified atmosphere with 5% CO2. THP-1 cells were pretreated with 12-O-tetradecanoylphorbol-13-acetate (PMA, 100 ng/ml, MCE, New Jersey, USA) for 24 h to differentiate into macrophages (hMφ).

### *Fn* infection and conditional medium collection

To determine the optimal MOI of *Fn*, cal27, SCC7, and RAW264.7 cells were infected with *Fn* at different MOI (MOI = 0, 10, 20, 50, 100, 200, 500, 1000) for 1d, 2d, and 3d. Subsequently, the CCK8 assay was performed using the CCK8 kit (Dojindo, Kumamoto, Japan). At the defined time points, the old medium was removed, and cells were incubated with 100 μl CCK8 solution (CCK8:medium = 1:10) at 37 °C for 2 h, protected from light. After incubation, absorbance at 450 nm was measured by a microplate reader. THP-1 cells were infected with *Fn* at MOI = 0, 10, 20, 50, 100, 200, then were stained by the live/dead cell staining kit (Solaibao, Beijing, China) according to the manufacturer’s instructions. The living cells (green) and dead cells (red) were visualized using a fluorescence microscope (Leica, Germany).

For conditioned medium acquisition, cal27, THP-1, SCC7, and RAW264.7 cells were incubated in serum-free medium with or without *Fn* at the optimal MOI for 48 h, then the conditioned medium was collected, centrifuged, filtered, and named as cal27-CM, (cal27+*Fn*)-CM, hMφ-CM, (hMφ + *Fn*)-CM, SCC7-CM, (SCC7+*Fn*)-CM, mMφ-CM and (mMφ + *Fn*)-CM. The conditioned medium was aliquoted and stored at −80 °C until use.

The experimental procedure for *E.coli* infection was similar to that described above.

### 5-Ethynyl-2′-deoxyuridine (EdU) assay

EdU assays were performed using the Cell-Light EdU Cell Proliferation Kit (RiboBio, Guangdong, China) following the manufacturer’s protocol. Cells were incubated with EdU buffer for 2 h, fixed with 4% PFA, and stained with Apollo Dye Solution. Next, DNA staining was conducted using Hoechst 33342. Images were obtained with a fluorescent microscope (Leica), and the number of EdU-positive cells was calculated by Image J.

### Colony formation assay

For colony formation assay, 1000 cal27 cells or 500 SCC7 cells were seeded in each well of six-well plates and subjected to different treatments for ten or five days. Colonies were fixed with PFA and stained with crystal violet (Solaibao). The number of clones that contain more than 50 cells was assessed.

### Wound healing assays

The cal27 cells or SCC7 cells were cultured in six-well plates to full confluence. 1 ml of blue pipette tips was used to generate a scratch. Cells were treated in different groups as indicated. Images of cells were taken at 0 and 24 h.

### Transwell migration assays

Cell migration assay was performed using transwell insert chambers (8-μm pore size, Corning). Cells were seeded in the upper well in 100 μl medium with 0.5% FBS. In the lower chamber, medium containing 0.5% FBS or 10% FBS was used as negative and positive controls, respectively, and the medium (0.5% FBS) with *Fn* or different treated conditioned medium were experimental groups. After 24 h, the cells that had migrated across the transwell membrane were fixed and stained with crystal violet. Images were randomly taken under a microscope. Every six fields were counted for each sample.

### RNA extraction and qRT-PCR

Total RNAs were extracted from cells using RNAiso Plus reagent (Takara, Japan). RNA was quantified using the Qubit RNA HS Assay Kit (Thermo Fisher Scientific) and reverse transcribed to cDNA using PrimeScript RT Master Mix (Takara). Then, qRT-PCR was performed using SYBR Premix Ex Taq (Yeasen, Shanghai, China) in the ROCHE LightCycler®480 System. Each reaction was tested triplicate in 10 µl of reaction volume. GAPDH was amplified as the internal reference, and relative abundance was calculated by the 2^-ΔΔCt^ method. Primer sequences are listed in Table 1.

**Table 1:** qRT-PCR primer sequences used in this study.

Gene	Forward primer	Reverse primer
Homo-Ki67	5’-TCAGACTCCATGTGCCTGAGA-3’	5’-CACATTGTCCTCAGCCTTCTTTG-3’
Homo-cyclin D1 (CCNDI)	5’-GAAGGAGACCATCCCCCTGA-3’	5’-GAAATCGTGCGGGGTCATTG-3’
Homo-vimentin	5’-AGGCGAGGAGAGCAGGATTT-3’	5’-AGTGGGTATCAACCAGAGGGA-3’
Homo-MMP9	5’-TCTGCCCGGACCAAGGATA-3’	5’-ACATAGGGTACATGAGCGCC-3’
Homo-ARG1	5’-TGACGGACTGGACCCATCTT-3’	5’-GGCTTGTGATTACCCTCCCG-3’
Homo-IL-10	5’- CCAGACATCAAGGCGCATGT-3’	5’-GATGCCTTTCTCTTGGAGCTTATT-3’
Homo-TGFβ1	5’-GCAACAATTCCTGGCGATACC-3’	5’-ATTICCCCTCCACGGCTCAA-3’
Homo-CD86	5’- CGACGTTTCCATCAGCTTGTC-3’	5’-CGCGTCTTGTCAGTTTCCAG-3’
Homo-INOS	5’- CGTGGAGACGGGAAAGAAGT-3’	5’-GACCCCAGGCAAGATTTGGA-3’
Homo-CXCL1	5’-TTGCCTCAATCCTGCATCCC-3’	5’-GTTGGATTTGTCACTGTTCAGCAT-3’
Homo-CXCL2	5’- TTGTCTCAACCCCGCATCG-3’	5’-CAGTTGGATTTGCCATTTTTCAG-3’
Homo-CCL2	5’-AATCAATGCCCCAGTCACCT-3’	5’-CTTCTTTGGGACACTTGCTGC-3’
Homo-CXCL5	5’-GACCACGCAAGGAGTTCATC-3’	5’-GGAGGCTACCACTTCCACCT-3’
Homo-CCL5	5’-CGAAAGAACCGCCAAGTGTG-3’	5’-CGGGTGGGGTAGGATAGTGA-3’
Homo-CXCL8	5’-CGGGTGGGGTAGGATAGTGA-3’	5’-GTTTTCCTTGGGGTCCAGACA-3’
Homo-CCL20	5’-TTATTGTGGGCTTCACACGG-3’	5’-TTGCGCACACAGACAACTTT-3’
Homo-GAPDH	5’-GCACCGTCAAGGCTGAGAAC-3’	5’-TGGTGAAGACGCCAGTGGA-3’
Mus-CXCL2	5’-CTGCCAAGGGTTGACTTCAAGA-3’	5’-CTTCAGGGTCAAGGCAAACT-3’
Mus-GAPDH	5’-TGTCTCCTGCGACTTCAACA-3’	5’-GGTGGTCCAGGGTTTCTTACT-3’

### Western blot

Cells or tumor tissues were lysed in RIPA lysis buffer and then centrifuged to obtain supernatant proteins. Protein concentrations were measured using a BCA assay (Solaibao). After denaturation at 95 °C for 5 min, proteins were separated by SDS-PAGE and transferred to PVDF membranes. The membranes were blocked with 5% milk powder and incubated with MMP9 (Proteintech, Wuhan, China, 10375-2-AP), Vimentin (Cohesion Biosciences, London, UK, CPA2228), CXCL2 (Abcam, MA, USA, ab12473, and Bioss, Shanghai, China, bs-1162R), Phospho-NF-κB p65 (Cell Signaling Technology, CST, MA, USA, 3033), NF-κB p65 (CST, 8242), Phospho-IκBα (CST, 2859), IκB-α (CST, 4814), Phospho-IKKα/β (CST, 2697), IKKα (CST, 11930), IKKβ (CST, 8943), GAPDH (Proteintech, HRP-60004), and β-actin antibody (Proteintech, 81115-1-RR) at 4 °C overnight. The following day, membranes were incubated with secondary antibodies for 1 h. Finally, the bands were visualized with ECL reagents.

### Immunofluorescence (IF)

Cells were seeded on 48-well plates and subjected to different treatments. The cells were fixed with 4% paraformaldehyde (PFA) for 20 min. After washing with PBS, the cells were blocked in 10% goat serum for 30 min, followed by incubation in the primary antibody at 4 °C overnight. Primary antibodies used were the following: against MMP9 (Proteintech, 10375-2-AP), Vimentin (Cohesion Biosciences, CPA2228), ki67 (Proteintech, 28074-1-AP), CD86 (CST, 91882, and Cohesion, CQA1883), CD206 (Abcam, ab64693), CXCL2 (Bioss, bs-1162R) and p65 (CST, 8242). The next day, wells were washed and incubated with fluorochrome-conjugated secondary antibodies for 1 h, then labeled with DAPI. Images were captured using a fluorescent microscope (Leica) or confocal microscope.

### Flow cytometry

For cell marker flow staining, cells were harvested and resuspended in FACS buffer (PBS contained 2% FBS). Then, the cells were blocked with Fc Receptor block solution (Biolegend, London, UK, Human TruStain FcX, 422301; anti-mouse CD16/32 antibody, 156603) and incubated with cell surface antibodies (CD86) on ice for 20 min. Following surface staining, cells were fixed and then permeabilized using a Fix/Perm buffer kit (BioLegend, 426803) and subsequently stained with intracellular antibodies (CD206). Cells were washed, resuspended in FACS buffer, and tested on CytoFlex (Beckman, USA). Flow antibodies were as follows: APC anti-human CD86 (Biolegend, 374207), FITC anti-human CD206 (Biolegend, 321103), APC anti-mouse CD206 (Biolegend, 141707), and PE anti-mouse CD86 (Biolegend, 129203).

### Enzyme-linked immunosorbent assay (ELISA)

Cells were treated according to different groups. The medium was then collected from different groups. After centrifugation, the supernatant was subjected to an ELISA assay (Lianke Biotechnology, Hangzhou, China) to analyze CXCL2 concentrations. The absorbance at 450 nm and 630 nm was measured by a microplate reader after adding the stop solution.

### Cell transfection

SiRNA-CXCL2 and its negative control were constructed by Hanheng Biotechnology (Hanheng, Shanghai, China). Cal27 cells and THP-1 cells were transfected with Lipofectamine 2000 (Lipo2000) (Invitrogen, California, USA) in opti-MEM (Gibco) according to the manufacturer’s protocols. Cells were harvested 48 h after transfection to assess transfection efficiency by PCR and western blot (Fig. S8). Cells transfected with si-CXCL2 were used for subsequent studies. The siRNA sequences are shown in Table 2.

**Table 2:** The sequences of siRNAs used in this study.

siRNA	Sense siRNA strand	Antisense siRNA strand
siRNA-CXCL2-1	5′-UGGGCAGAAAGCUUGUCUCAAdTdT-3′	5′-UUGAGACAAGCUUUCUGCCCAdTdT-3′
siRNA-CXCL2-2	5′-GCAUCGCCCAUGGUUAAGAAAdTdT-3′	5′-UUUCUUAACCAUGGGCCAUGCdTdT-3′
siRNA-CXCL2-3	5′-GCAUCGCCCAUGGUUAAGAAAdTdT-3′	5′-UUUCUUAACCAUGGGCCAUGCdTdT-3′
Control siRNA	5′-UUCUCCGAACGUGUCACGUdTdT-3′	5′-ACGUGACACGUUCGGAGAAdTdT-3′

### OSCC mouse model

All animal studies were approved by the Ethics Committee of the Hospital of Stomatology, Shandong University (No. 20221148). Five-week-old male C3H mice were obtained from Weitong Lihua Experimental Animal Center (Beijing, China) and cultured in specific-pathogen-free (SPF) facilities. After one week of adaptive rearing, 10^6^ SCC7 cells were subcutaneously injected into the backs of C3H mice to establish the SCC7 tumor-bearing model. Mice were randomly divided into two groups (five animals in each group): (i) Control; (ii) *Fn* infection. One week following the SCC7 injection, the *Fn* was given by multi-point intratumoral injection, repeated every three days (Fig. 6A). The tumor volume was also monitored every three days with calipers and was calculated using the following formula: Volume = (width)^2^ × length/2.

The mice were sacrificed on day 19 post-SCC7 injection, and tumors were harvested, photographed, and weighed. Portions of tumor tissue were fixed in 4% PFA and embedded in paraffin. Another portion was used for flow staining. Single-cell suspensions were prepared by mechanical dispersion and enzymatic digestion of mouse tumor tissues (Fig. 6D). Cells were stained using the method described in the “Flow cytometry” section. Antibodies used are as follows: FITC anti-mouse F4/80 (Biolegend, 123107), APC anti-mouse CD206 (Biolegend, 141707), and PE anti-mouse CD86 (Biolegend, 129203).

CXCR2 inhibitor SB225002 was dissolved at 1% DMSO, 20% polyethylene glycol 400, 5% tween 80, and 74% saline. One week after tumor implantation, when the tumor volume reached 50–100 mm^3^, mice were randomly divided into four groups (five animals in each group): (i) Control; (ii) *Fn*; (iii) SB225002; (iv) *Fn* + SB225002. SB225002 was administered at 10 mg/kg by intraperitoneal (i.p.) injection every 2 days. The control and *Fn* group received solvent (1% DMSO, 20% PEG 400, and 5% Tween 80).

### Immunohistochemical (IHC) staining and IF staining of tissue sections

Paraffin sections were dewaxed, rehydrated, and repaired with an antigen repair solution. IHC analysis was performed by following the kit instructions (Zhongshan Golden Bridge Biotechnology, Beijing, China). Primary antibodies for IHC included ki67 (Proteintech, 28074-1-AP), p65 (CST, 8242), CXCL2 (Bioss, bs-1162R), CD86 (Abcam, ab220188) and CD206 (Abcam, ab64693).

After antigen repair, the IF staining steps of tissue sections were similar to the cell IF above. The applied primary antibodies were p65 (CST, 8242), CD86 (Abcam, ab220188), and CD206 (Abcam, ab64693).

### Statistical analysis

Statistical analyses were performed using GraphPad Prism 8.0. Differences between groups were tested using the student’s t-test or one-way ANOVA. Each experiment was repeated three times in three replicates. All experimental data were presented as the mean ± S.D. Statistical significance was established at two-sided *P* < 0.05.

### Supplementary information

Original western blots

Supplementary material

## Data Availability

All the data supporting the findings of this study are presented in the paper or the Supplementary Materials. The datasets and materials employed during the research can be obtained from the corresponding author upon a reasonable request.
