# Supplementary material for: The role of CXCL2-mediated crosstalk between tumor cells and macrophages in Fusobacterium nucleatum-promoted oral squamous cell carcinoma progression
Source: Cell Death Dis. 2024 Apr 18;15(4):277. doi: 10.1038/s41419-024-06640-7 (PMC11026399; doi:10.1038/s41419-024-06640-7)

Original Western blots

Uncropped membranes for Fig. 2G

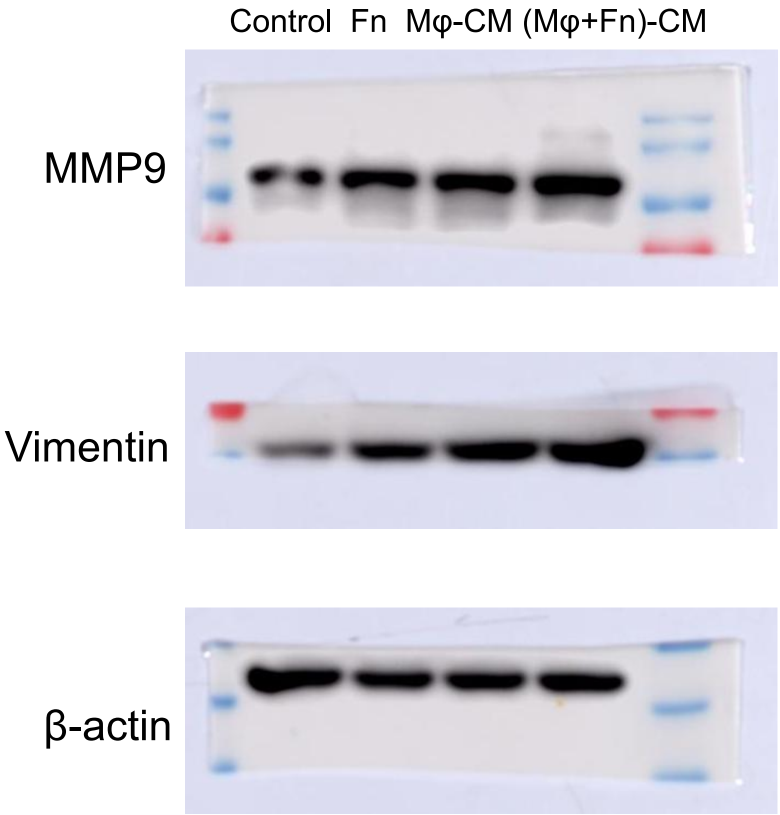

Uncropped membranes for Fig. 5A

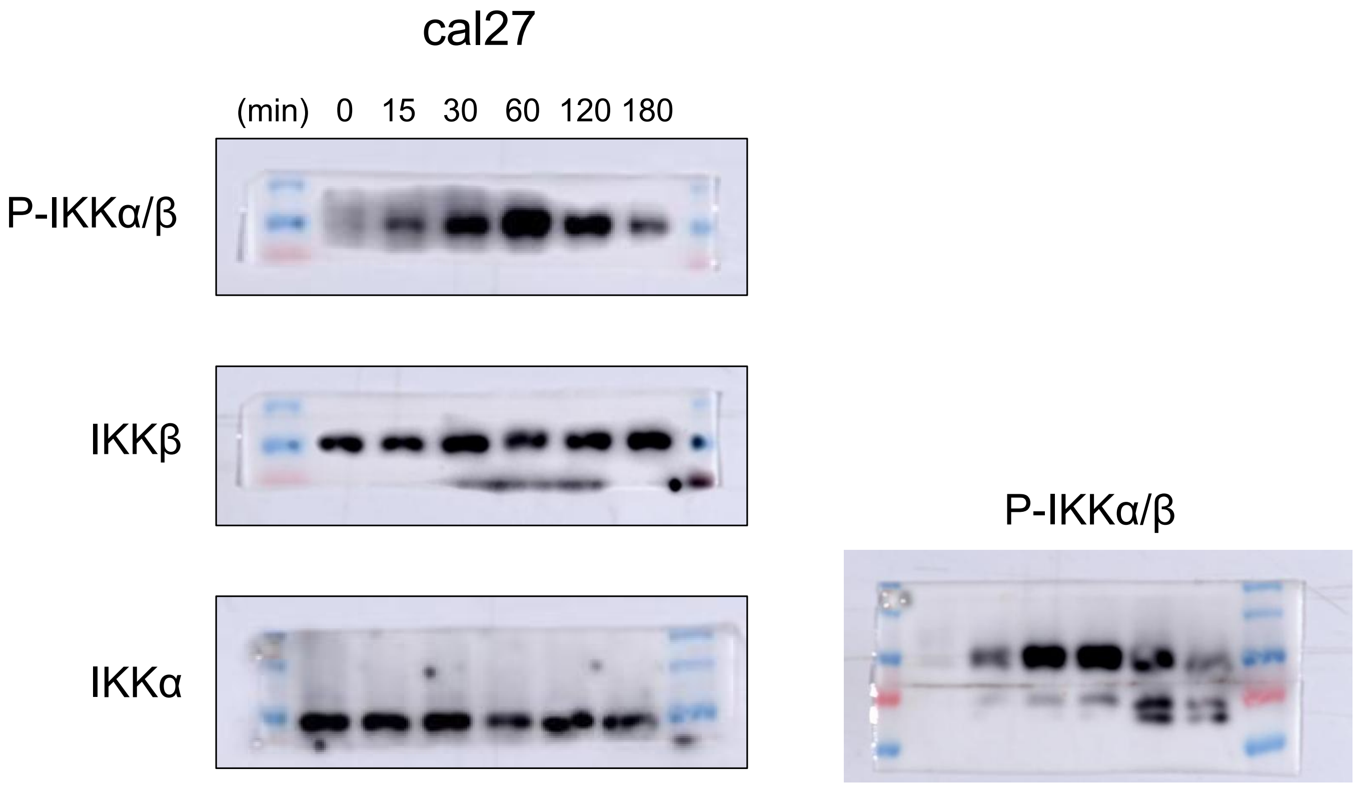

The IKKα band comes from the same membrane as the one on the right.

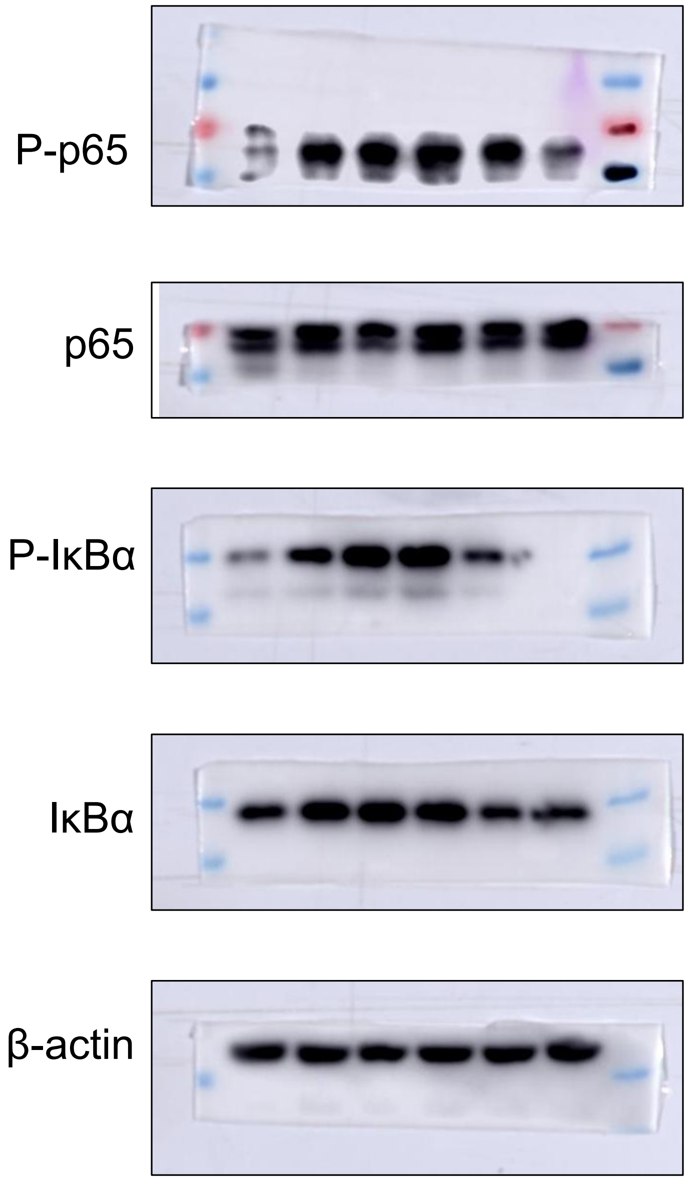

Uncropped membranes for Fig. 5A

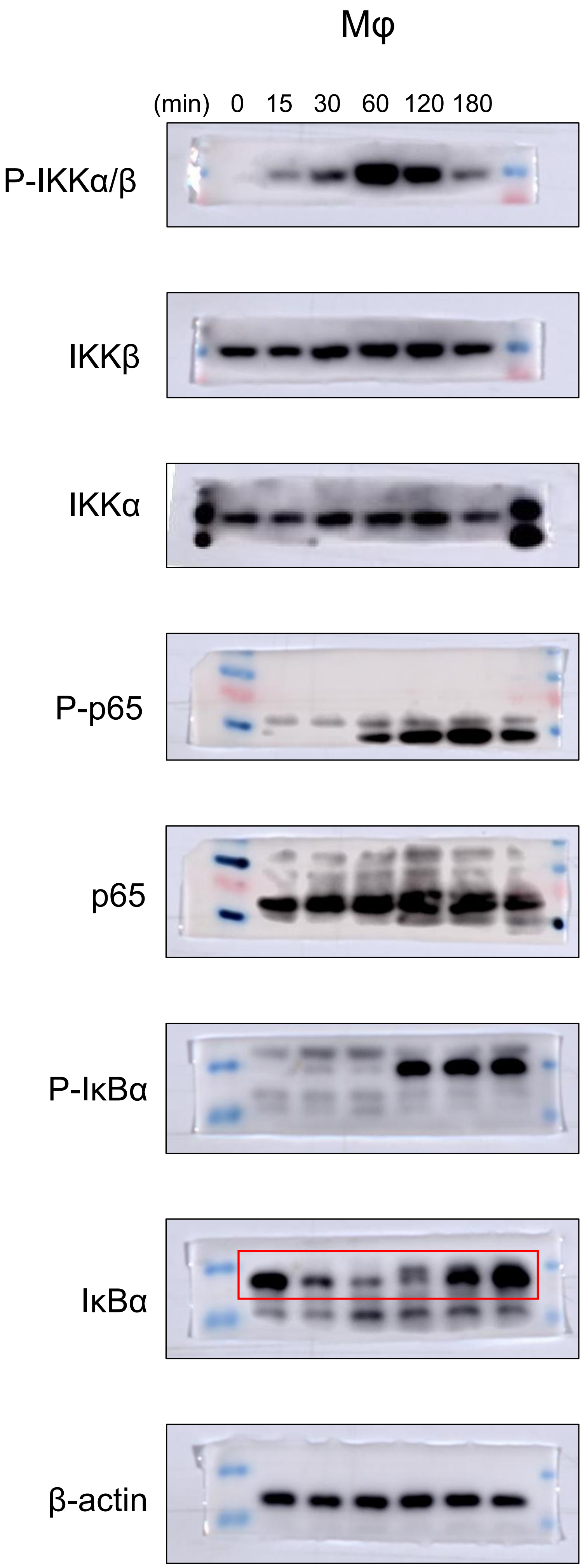

Uncropped membranes for Fig. 5C

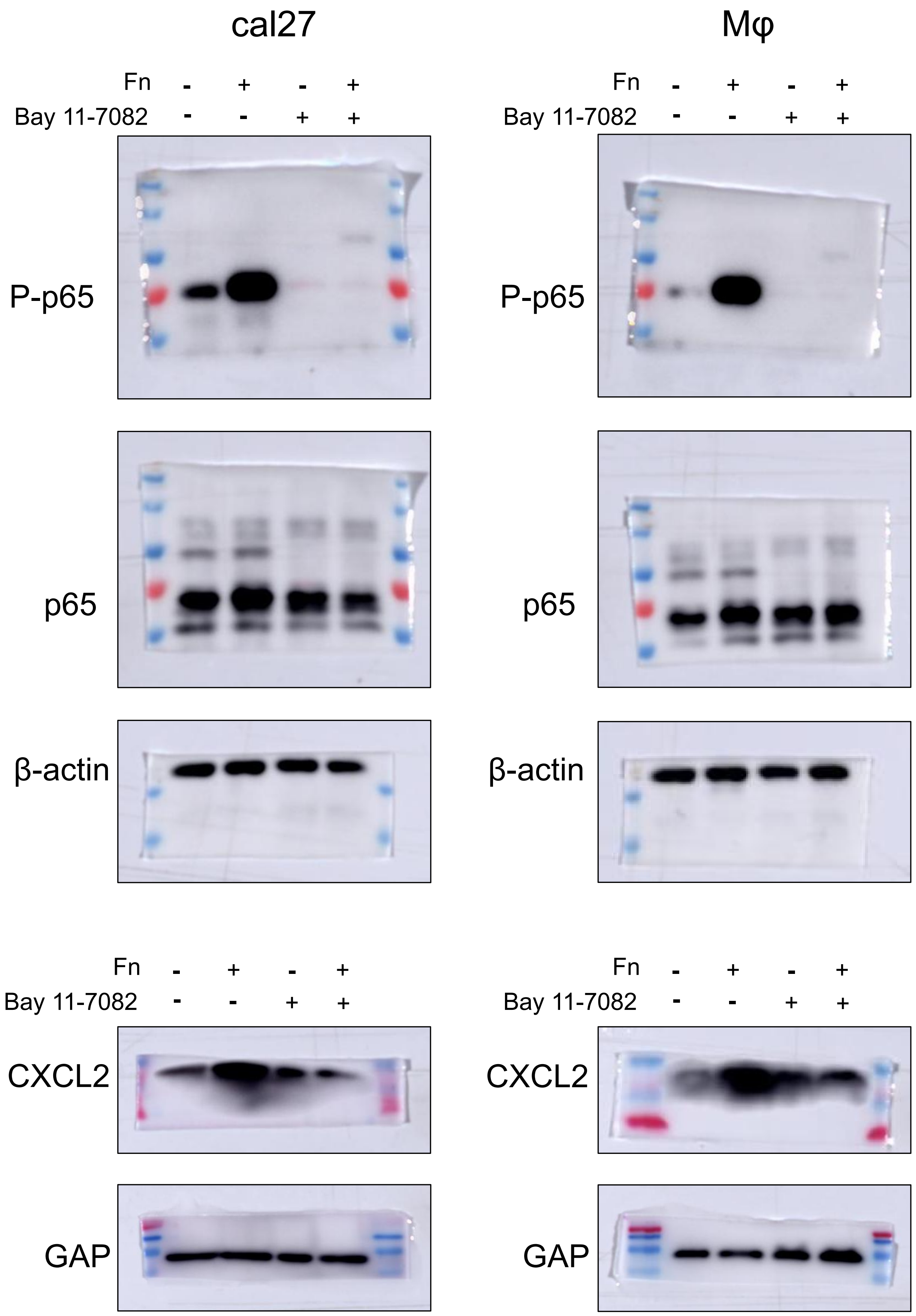

Uncropped membranes for Fig. S8A

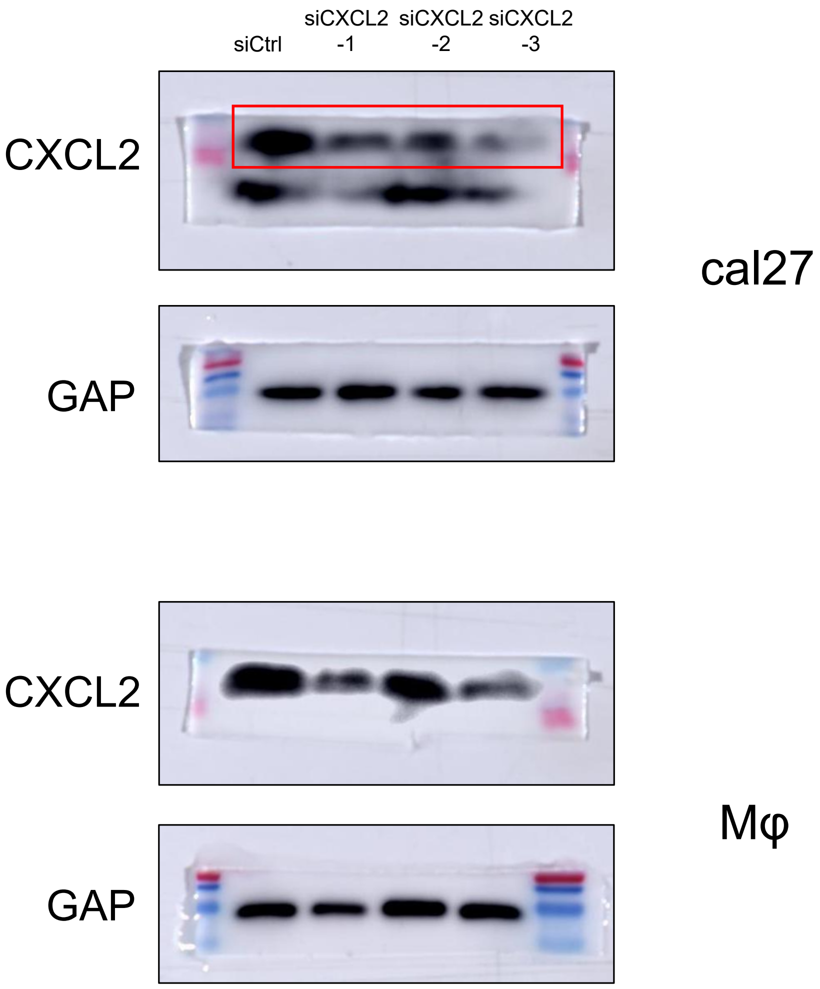

Uncropped membranes for Fig. S11

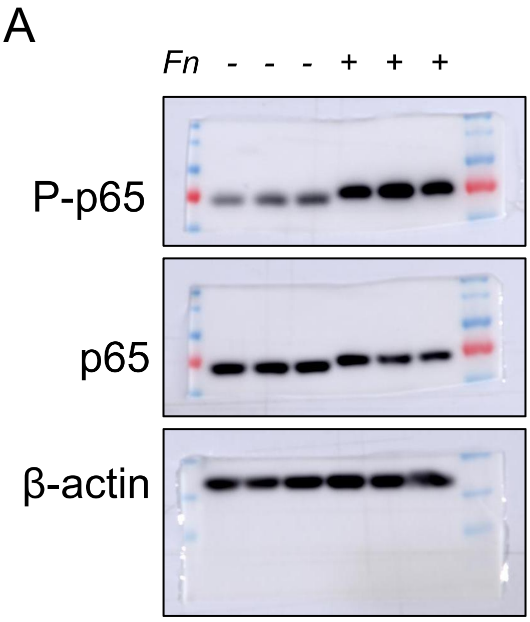

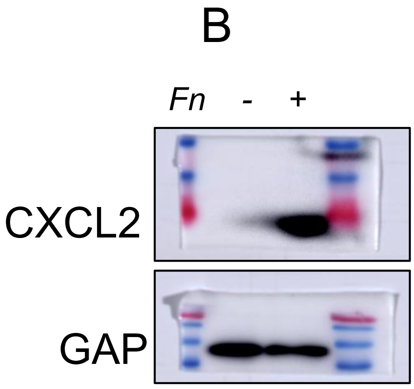

Supplement: Supplementary file 1 — Original western blots [file 41419_2024_6640_MOESM1_ESM.pdf]
