# Supplementary material for: The role of CXCL2-mediated crosstalk between tumor cells and macrophages in Fusobacterium nucleatum-promoted oral squamous cell carcinoma progression
Source: Cell Death Dis. 2024 Apr 18;15(4):277. doi: 10.1038/s41419-024-06640-7 (PMC11026399; doi:10.1038/s41419-024-06640-7)
Supplement: Supplementary file 2 — Supplementary material [file 41419_2024_6640_MOESM2_ESM.pdf]

# Supplementary figures:

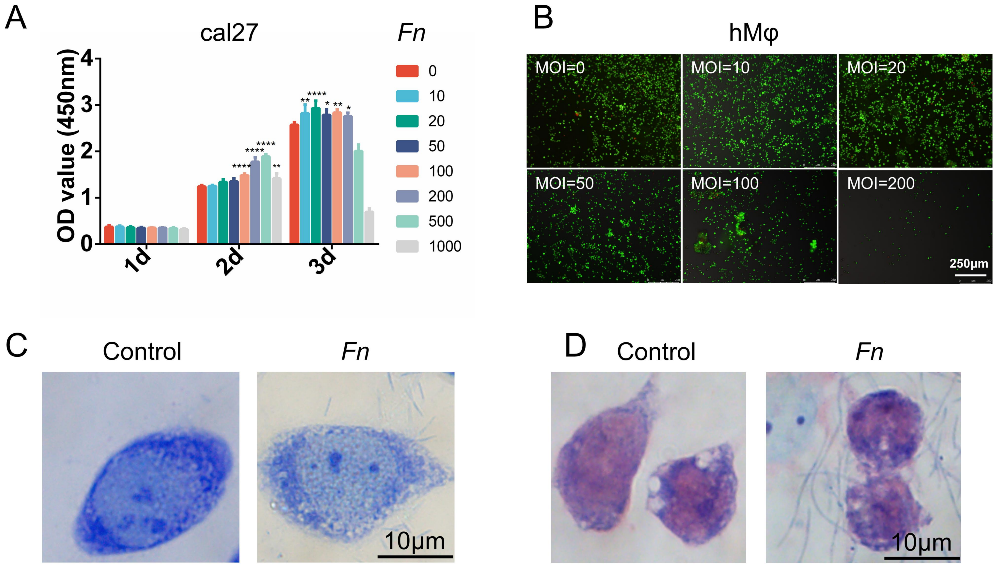


**Fig. S1 Determination of the optimal multiplicities of infection (MOI). A** The CCK8 method was used to detect the effect of *Fn* infection with different MOI on the viability of cal27 cells at different times. **B** Live/Dead staining results of THP-1 cells (hMφ) incubated with *Fn* at varying MOI. **C, D** *Fn* infection was stained using the Diff-Quik system in cal27 cells (C) and THP-1 cells (D). vs control group (MOI=0): * *P* < 0.05, ** *P* <0.01, *** *P* < 0.001, and **** *P* < 0.0001, ns no significant.

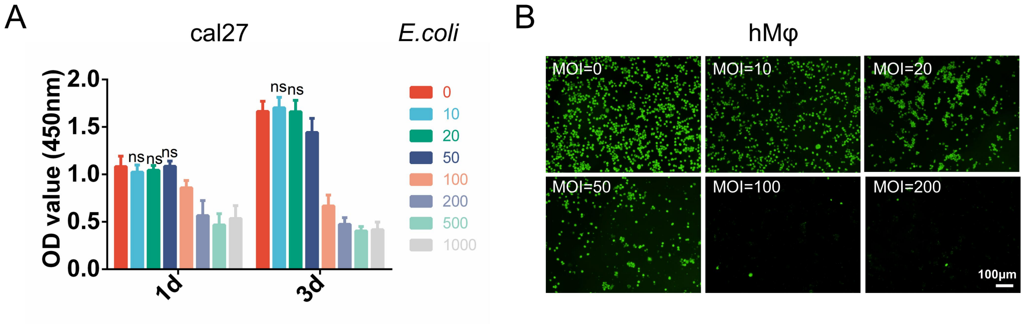


**Fig. S2 *E.coli* cannot promote the proliferation of cal27 cells and the adhesion of THP-1 cells (hMφ). A** The CCK8 method was used to detect the effect of *E.coli* infection with different MOI on the viability of cal27 cells at different times. **B** Live/Dead staining results of THP-1 cells (hMφ) incubated with *E.coli* at varying MOI. vs control group (MOI=0): * *P* < 0.05, ** *P* <0.01, *** *P* < 0.001, and **** *P* < 0.0001, ns no significant.

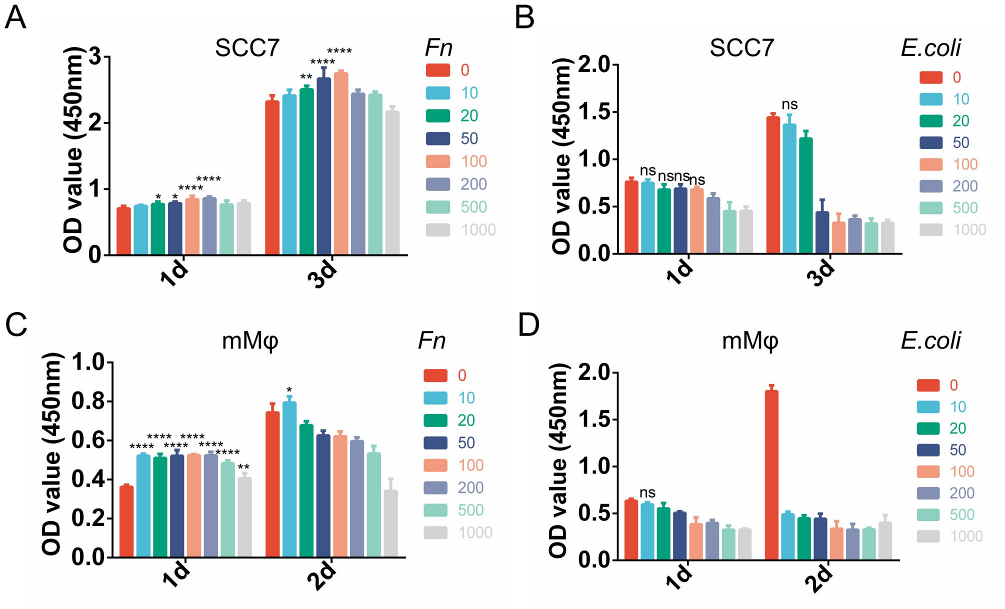


**Fig. S3 Determination of the optimal MOI. A-D** The CCK8 method was used to detect the effect of *Fn* (A and C) or *E.coli* (B and D) infection with different MOI on the viability of SCC7 and RAW264.7 cells (mMφ) at different times. vs control group (MOI=0): * *P* < 0.05, ** *P* <0.01, *** *P* < 0.001, and **** *P* < 0.0001, ns no significant.

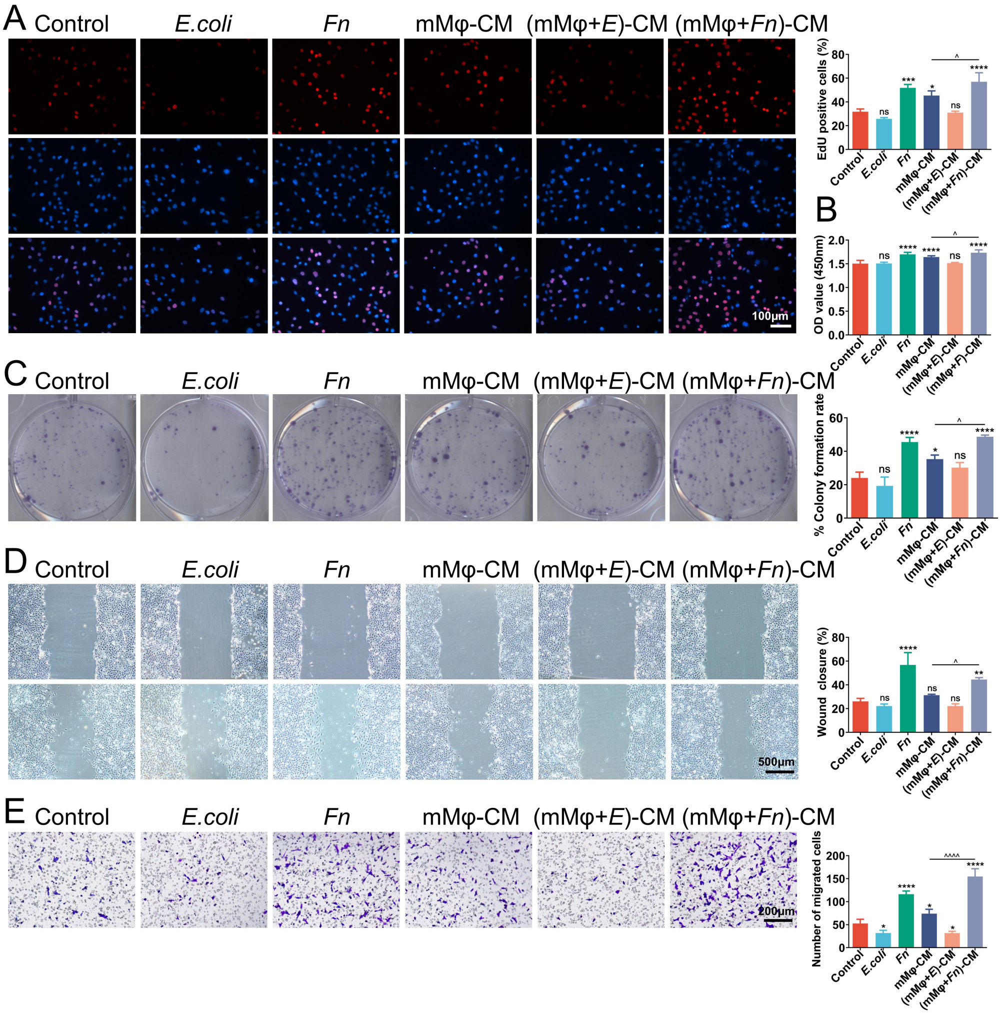


**Fig. S4 Both *Fn* and macrophages contribute to the proliferation and migration of SCC7 cells, and *Fn*-challenged macrophages amplify this pro-tumor effect. A-C** The proliferation ability of SCC7 cells treated with *Fn*, *E.coli*, mMφ-CM, (mMφ+*E*)-CM, and (mMφ+*Fn*)-CM was assessed using Edu assays (A), CCK8 assays (B), and colony formation assays (C). **D-E** The migratory capacity of SCC7 cells was detected by wound healing assay (D) and transwell assay (E). vs control group: * $P < 0.05$, ** $P < 0.01$, *** $P < 0.001$, and **** $P < 0.0001$, ns no significant. Comparison between two groups: ^ $P < 0.05$, ^^ $P < 0.01$, ^^^ $P < 0.001$, and ^^^^ $P < 0.0001$, ns no significant.

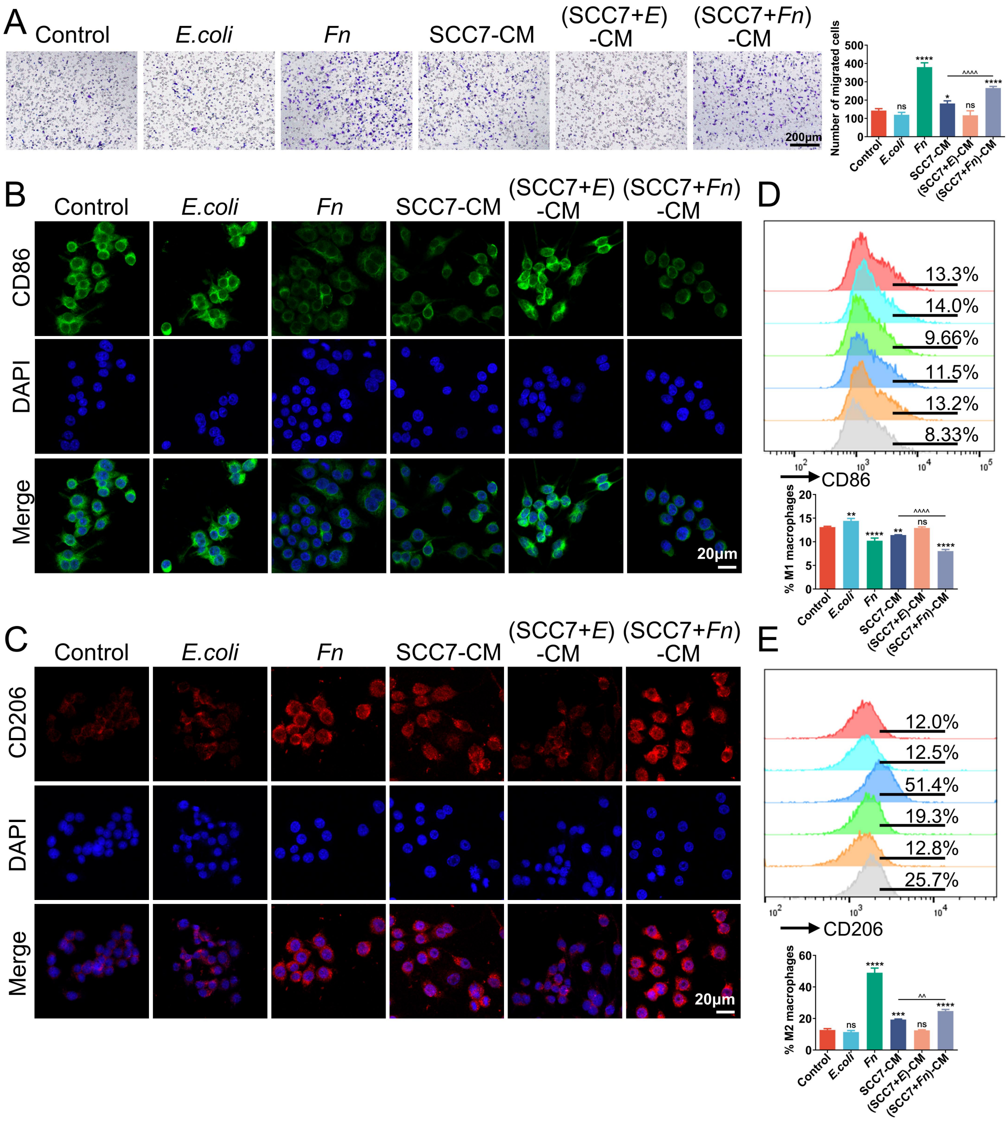


**Fig. S5 Both *Fn* and SCC7 cells recruit macrophages and promote M2 macrophage polarization, and *Fn*-challenged SCC7 cells augment the effects. A** Transwell assays assessed the capacity of *Fn* and SCC7 cell conditioned medium to recruit RAW264.7 cells. **B and C** Macrophage polarization markers in RAW264.7 cells were measured by IF assays. **D and E** Flow cytometry was performed to assess the polarization of RAW264.7 cells. vs control group: * $P < 0.05$, ** $P < 0.01$, *** $P < 0.001$, and **** $P < 0.0001$, ns no significant. Comparison between two groups: ^ $P < 0.05$, ^^ $P < 0.01$, ^^^ $P < 0.001$, and ^^^^ $P < 0.0001$, ns no significant.

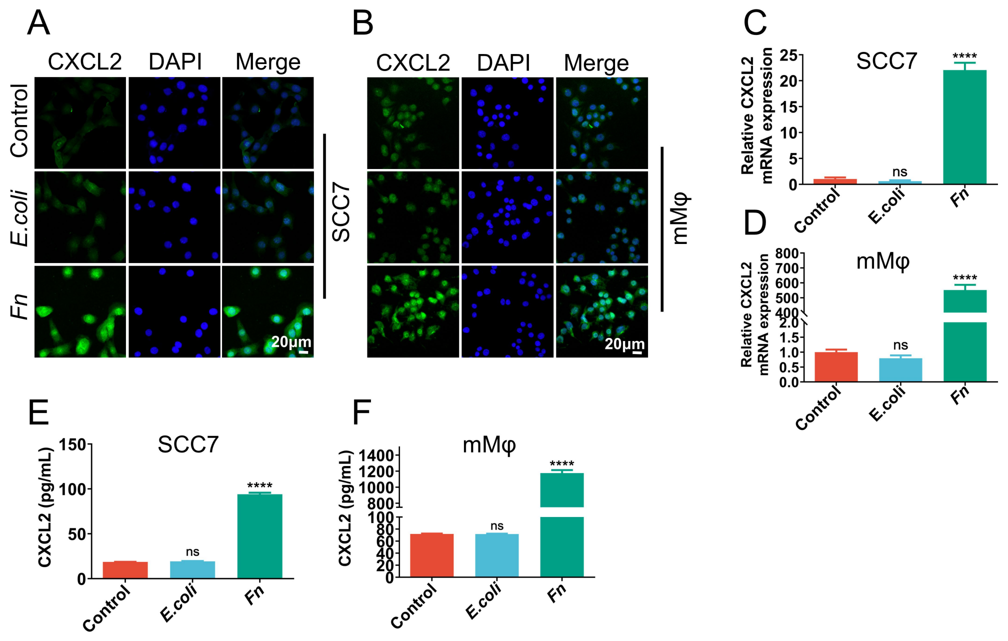


**Fig. S6 *Fn* upregulates CXCL2 expression and secretion in SCC7 and RAW264.7 cells. A, B** IF staining for CXCL2 protein expression in SCC7 cells (A) and RAW264.7 cells (B). **C, D** qRT-PCR analysis shows the mRNA expression of CXCL2 in SCC7 cells (C) and RAW264.7 cells (D). **E, F** ELISA results of CXCL2 secretion in SCC7 cells (E) and RAW264.7 cells (F). vs control group: * $P < 0.05$, ** $P < 0.01$, *** $P < 0.001$, and **** $P < 0.0001$, ns no significant.

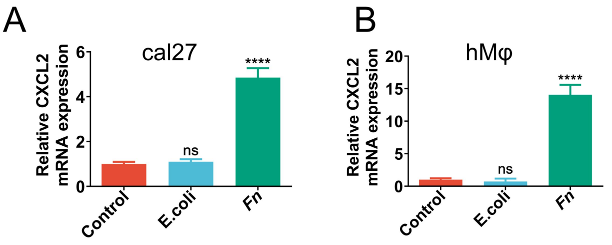


**Fig. S7 *E.coli* had no effect on the expression of CXCL2. A** qRT-PCR assay of cal27. **B** qRT-PCR assay of THP-1 derived macrophages. vs control group: * $P < 0.05$, ** $P < 0.01$, *** $P < 0.001$, and **** $P < 0.0001$, ns, no significant.

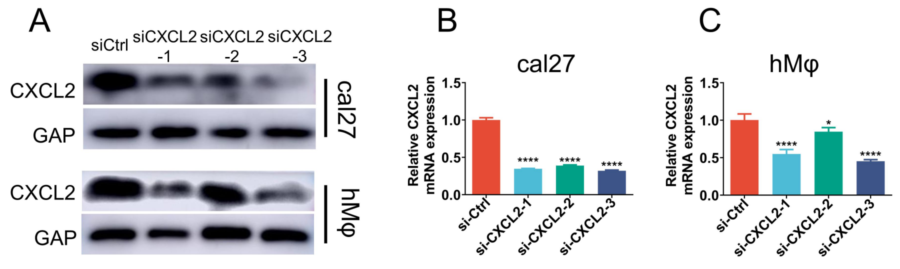


**Fig. S8 Verification of siRNA CXCL2 knockdown efficiency. A** Western blot

analysis validation for CXCL2 knockdown in cal27 cells and THP-1 cells. **B, C** qRT-PCR analysis showing CXCL2 knockdown in cal27 cells (B) and THP-1 cells (C). vs control group: * $P < 0.05$, ** $P < 0.01$, *** $P < 0.001$, and **** $P < 0.0001$, ns, no significant.

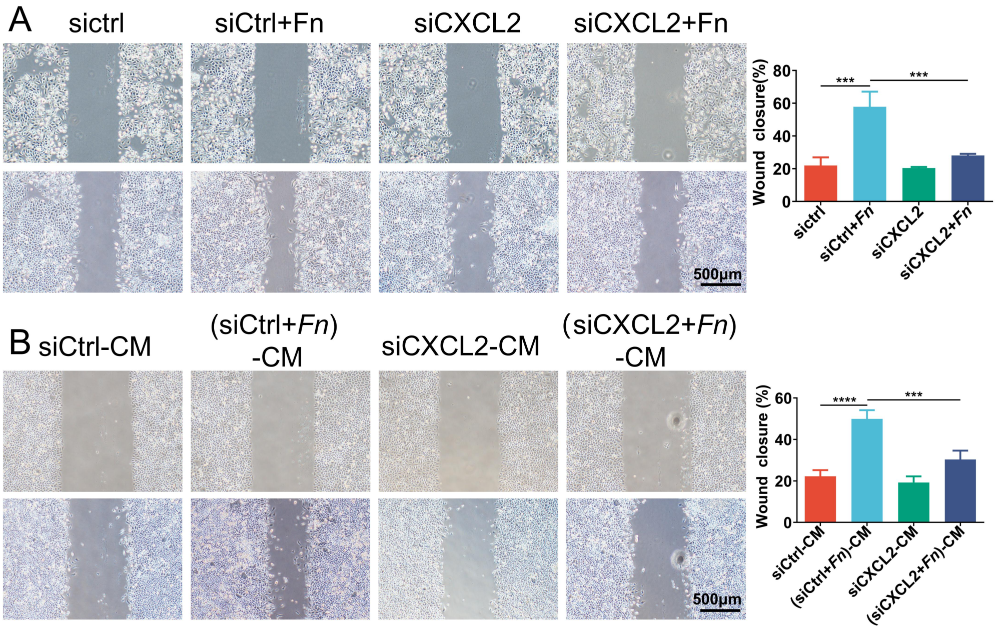


**Fig. S9 The impact of CXCL2 deficiency on *Fn*-induced cal27 cell migration. A** Pretreated with CXCL2 siRNA or control siRNA, cal27 cells were incubated with or without *Fn* for 24h. Cell migration of transfected cells was detected by a scratch assay. **B** THP-1 cells were pretreated with CXCL2 or control siRNA, followed by incubation with or without *Fn* for 24 h. The conditioned medium was collected to determine its impact on the migration of cal27 cells. * $P < 0.05$, ** $P < 0.01$, *** $P < 0.001$, and **** $P < 0.0001$, ns, no significant.

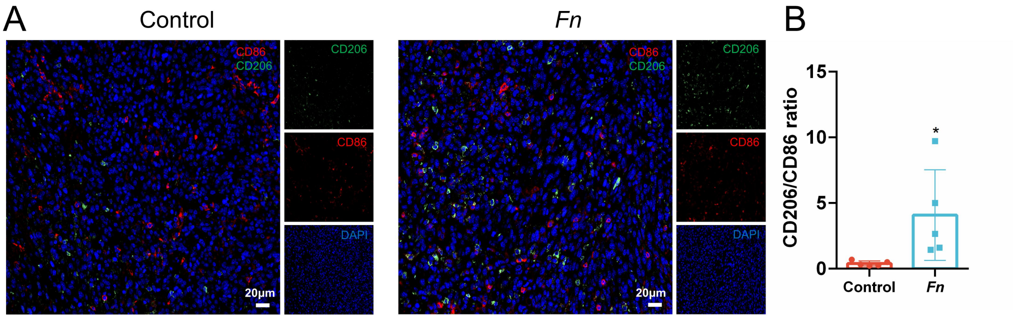


**Fig. S10 The CD206/CD86 ratio is significantly increased in the *Fn* group. A** Representative images of IF staining for CD86 (red) and CD206 (green) in the tumor tissues. **B** the ratio of CD206/CD86. vs control group: * $P < 0.05$, ** $P < 0.01$, *** $P < 0.001$, and **** $P < 0.0001$, ns, no significant.

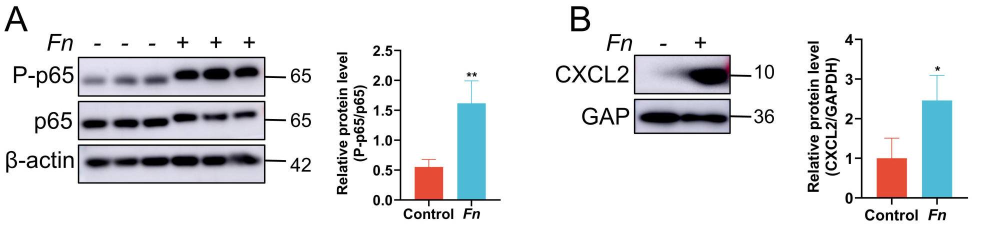


**Fig. S11 Protein expression in mouse tumor tissues. A, B** Protein lysates extracted from tumor tissues of mice were assayed with western blot. vs control group: * *P* < 0.05, ** *P* <0.01, *** *P* < 0.001, and **** *P* < 0.0001, ns, no significant.

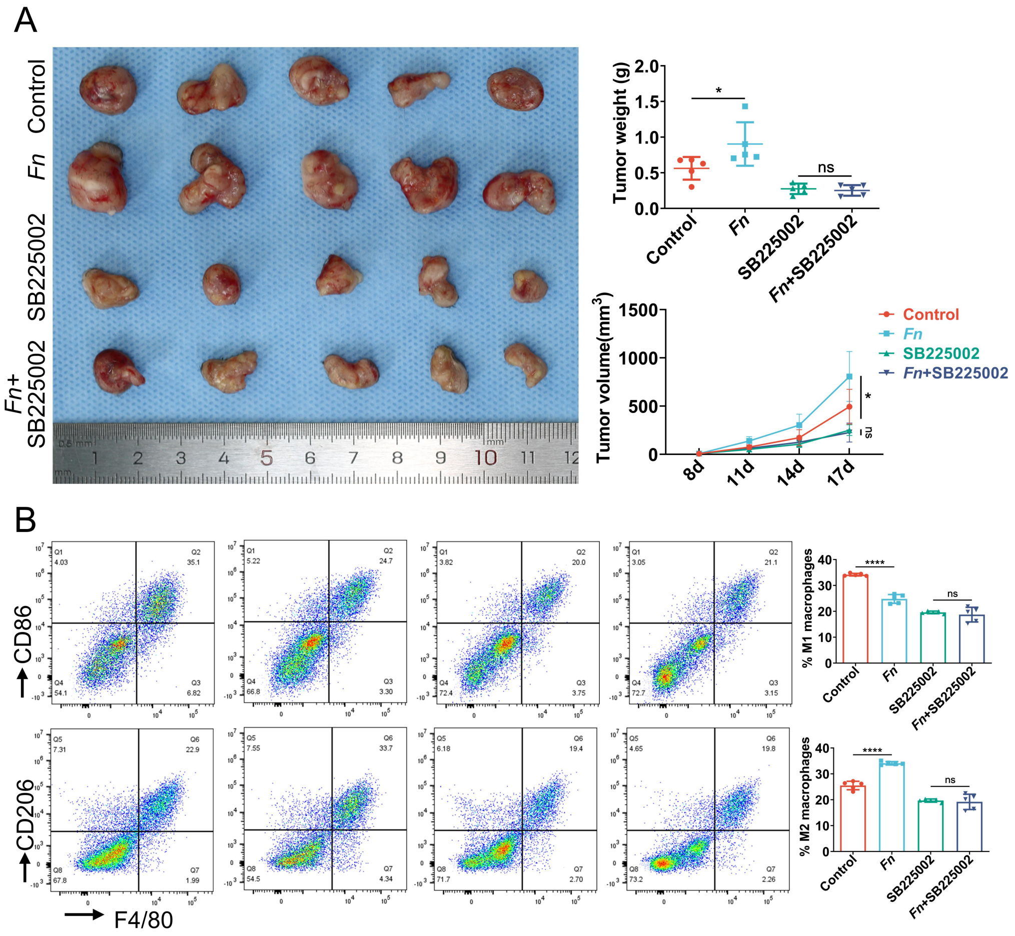


**Fig. S12 CXCL2-CXCR2 blocking reverses the tumor-promoting effect of the *Fn*. A** Photographs of tumors and tumor growth curves and weights. **B** Representative flow cytometry plots of tumor infiltrating M1 macrophages (F4/80+ CD86+) and M2 macrophages (F4/80+ CD206+). * *P* < 0.05, ** *P* <0.01, *** *P* < 0.001, and **** *P* < 0.0001, ns, no significant.
